# Identification of cell barcodes from long-read single-cell RNA-seq with BLAZE

**DOI:** 10.1186/s13059-023-02907-y

**Published:** 2023-04-06

**Authors:** Yupei You, Yair D. J. Prawer, Ricardo De Paoli-Iseppi, Cameron P. J. Hunt, Clare L. Parish, Heejung Shim, Michael B. Clark

**Affiliations:** 1 School of Mathematics and Statistics/Melbourne Integrative Genomics, The University of Melbourne, Parkville, VIC 3010 Australia; 2 Centre for Stem Cell Systems, Department of Anatomy and Physiology, The University of Melbourne, Parkville, VIC 3010 Australia; 3 The Florey Institute of Neuroscience and Mental Health, The University of Melbourne, Parkville, VIC 3010 Australia

## Abstract

Long-read single-cell RNA sequencing (scRNA-seq) enables the quantification of RNA isoforms in individual cells. However, long-read scRNA-seq using the Oxford Nanopore platform has largely relied upon matched short-read data to identify cell barcodes. We introduce BLAZE, which accurately and efficiently identifies 10x cell barcodes using only nanopore long-read scRNA-seq data. BLAZE outperforms the existing tools and provides an accurate representation of the cells present in long-read scRNA-seq when compared to matched short reads. BLAZE simplifies long-read scRNA-seq while improving the results, is compatible with downstream tools accepting a cell barcode file, and is available at https://github.com/shimlab/BLAZE**.**

**Supplementary Information:**

The online version contains supplementary material available at 10.1186/s13059-023-02907-y.

## Background

Single-cell transcriptomics has become a widely accessible and popular means of profiling gene expression at single-cell resolution. The applications of single-cell RNA sequencing (scRNA-seq) are broad, ranging from identifying cell and tissue types, tracking developmental trajectories, and assessing system heterogeneity [1]. However, short-read scRNA-seq methodologies lack the ability to accurately identify RNA isoforms. Droplet-based platforms such as the popular 10x platform [2] are restricted to sequencing the 3′ or 5′ ends of transcripts, providing accurate gene counts but little information on RNA splicing or the RNA isoforms expressed in each cell [3]. Alternative methods, such as Smart-seq3, sequence all parts of transcripts but are still constrained by short sequencing read lengths, which largely prevents the accurate reconstruction of RNA isoforms longer than 1 kb [4].

The recent development of long-read single-cell sequencing methods has laid the foundation for a more in-depth analysis of isoforms for single cells [5]. Long-read scRNA-seq methods have been developed using both the PacBio and Oxford Nanopore Technologies (ONT) platforms, allowing for the discovery and quantification of full-length RNA isoforms in single cells [6–19].

Two key steps in enabling scRNA-seq analysis are the identification of cell barcodes, which denote which cell a read is from, and unique molecular identifiers (UMIs), which allow the removal of PCR duplicates and more accurate counting of gene and isoform expression. A limitation of most long-read scRNA-seq methodologies is that they require matched short-read scRNA-seq for the identification of cell barcodes and/or UMIs, particularly those using nanopore sequencing due to its higher error rate [8, 9, 12, 14–17, 19]. The addition of matched short-read data adds technical complications for library construction and significantly increases both the time and cost needed to produce these datasets. Furthermore, the requirement for matched short-read data can also greatly decrease the proportion of usable long-reads [9]. Other methods for nanopore long-read scRNA-seq have been reported that do not require the addition of matched short reads. However, these methods are either very low throughput [10], require bespoke reagents and are incompatible with existing 10x workflows [13], or trade higher accuracy for lower read depth [18]. Therefore, a method which requires only nanopore long reads and is compatible with existing workflows is required [15]. Recently, ONT released the Sockeye pipeline (https://github.com/nanoporetech/Sockeye) to perform long read-only scRNA-seq analysis, including barcode and UMI identification. However, the performance of Sockeye is yet to be determined.

Here, we introduce Barcode identification from Long-reads for AnalyZing single-cell gene Expression (BLAZE), which accurately identifies 10x cell barcodes using only nanopore long-read scRNA-seq data. In combination with the existing FLAMES pipeline [15], BLAZE eliminates the requirement for matched short-read scRNA-seq, simplifying long-read scRNA-seq workflows, reducing sequencing costs, and producing improved results. We show that BLAZE performs well across different sample types, sequencing depths, and sequencing accuracies and outperforms other barcode identification tools such as Sockeye. We designed BLAZE to seamlessly integrate with the existing FLT-seq—FLAMES pipeline which performs UMI calling, read assignment, and mapping to enable the identification and quantification of RNA isoforms and their expression profiles across individual cells and cell types. Taken together, BLAZE provides a cheaper, simpler, and more accurate means to profile transcript-level changes in long-read scRNA-seq datasets.

## Results

### Single-cell barcode identification with BLAZE

**Fig. 1:**
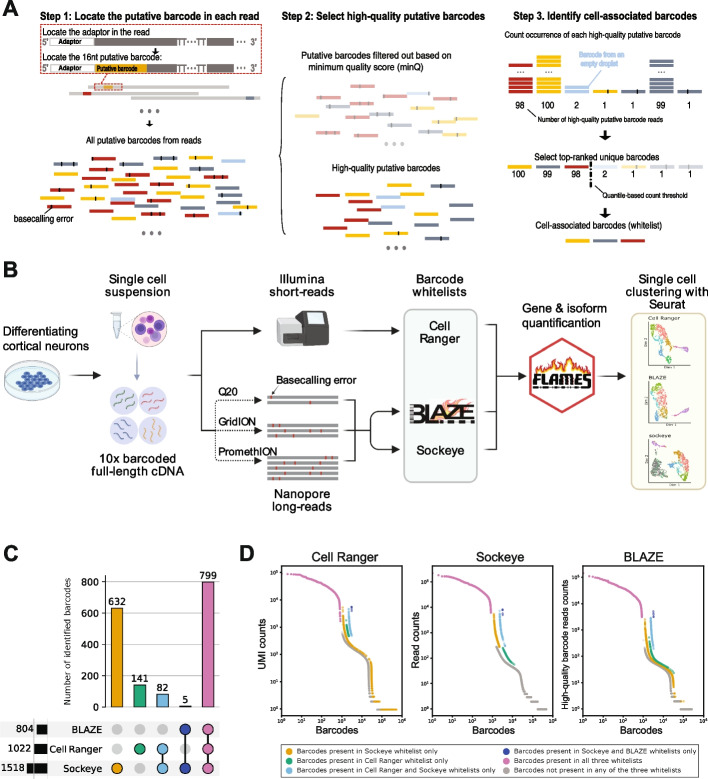
Experimental overview and comparison of identified cell barcodes. **A** BLAZE Workflow. Step 1: locate putative barcodes by first locating the adaptor in each read. Putative barcodes include those originating from different cells and empty droplets. In the schematic, putative barcodes with the same color come from the same original cell/droplet. Black blocks on putative barcodes represent basecalling errors. Step 2: select high-quality putative barcodes. Bases representing sequencing errors tend to have low quality scores. Putative barcodes with minQ < 15 are filtered out (faded in the figure) and the majority of the remaining putative barcodes are expected to have no errors. Step 3: identify cell-associated barcodes. BLAZE counts and ranks unique high-quality putative barcodes and outputs a list of cell-associated barcodes whose counts pass a quantile-based threshold.** B** Schematic of experimental design. Human induced pluripotent stem cells (hiPSC) undergoing cortical neuronal differentiation were dissociated into a single-cell suspension and processed to generate single-cell full-length cDNA. Full-length cDNA was sequenced using both short and long-read methods and barcode whitelists generated using Cell Ranger, BLAZE, and Sockeye followed by gene and isoform quantification and clustering. Three nanopore sequencing runs were performed on the same cDNA sample, a higher-depth PromethION run, a lower-depth GridION run, and a higher accuracy run using the Q20 protocol on the GridION. **C** Barcode upset plot comparing the different whitelists. The bar chart on the left shows the total number of barcodes found by each tool. The bar chart on the top shows the number of barcodes in the intersection of whitelists from specific combinations of methods. The dots and lines underneath show the combinations. The colors of the combinations are used to distinguish barcodes in Fig. 1D.** D** Barcode rank plot. Unique barcodes are ranked based on the counts output by each method and colored by which method(s) included each barcode in their barcode whitelist(s). The colors for different combinations of methods follow those in **C**, and barcodes not included in any of the whitelists are in gray. Cell Ranger short-read counts, Sockeye long-read counts, and BLAZE long-read counts shown on left, middle, and right knee plots, respectively. Sockeye and BLAZE analyze the same dataset. Cell Ranger analyzes counts from a short-read library, deriving from the same original cDNA. Unique barcodes are ranked on the *x*-axis based on the number of reads/unique molecules observed for each (*y*-axis). Shifts on the *x*-axis are intentionally added to make the dots with different colors non-overlapping. Note that these three methods generate counts in different ways so the three plots have different *y*-axis labels

We designed BLAZE for the accurate identification of cell barcodes from Oxford Nanopore long-read libraries generated using the 10x single-cell 3′ gene expression profiling procedure. To enable cell barcode identification from nanopore reads despite their higher error rate, BLAZE performs a three-step procedure (Fig. 1A, see the “Materials and methods” section for further details). First, BLAZE identifies the likely position of the cell barcode and extracts the putative barcode sequence. The 16-nt barcode and the 10–12-nt UMI are located between the adapter and polyT sequences. BLAZE locates the cell barcode in each read by identifying the probable adaptor and polyT regions. The 16-nt sequence immediately downstream of the adaptor is defined as the “putative barcode.” BLAZE discards putative barcodes that do not appear in the list of all possible 10x barcodes because these cannot represent true barcodes. Next, BLAZE selects high-quality putative barcodes whose sequences are less likely to contain base-calling errors. Specifically, BLAZE filters out barcodes with a minimum quality score (denoted as “minQ”) of less than 15 across the 16 bases that comprise the putative barcode (Additional file 1: Fig. S1). Finally, BLAZE counts the occurrence of each unique high-quality barcode, ranks them based on those counts, and selects the top-ranked ones as barcodes likely associated with cells using a quantile-based threshold.

A significant proportion of putative barcodes are expected to be error-free, despite the ~ 4–5% (or ~ 2% with higher-accuracy Q20 protocols) median error rate for nanopore reads (Table 1). With sufficient per-cell sequencing depth, this means each cell should be supported by a large number of high-quality putative barcodes. Therefore, highly supported barcodes likely represent true cells while poorly-supported barcodes likely represent sequencing errors and/or barcodes associated with empty droplets (Fig. 1A). The output of BLAZE is a list of unique cell-associated barcodes (referred to as barcode whitelist) that can be input into downstream gene and isoform quantification software in place of a whitelist generated from matched short-read sequencing.

**Table 1:** Summary statistics for long-read single-cell datasets

Dataset ID	Sequencing platform	Sequencing kit	Total reads	Pass reads	Mean reads per cell^a^	Median accuracy of pass reads (%)	Useable^b^ reads with Cell Ranger	Useable reads with BLAZE	Useable reads with Sockeye
PromethION	PromethION	SQK-LSK110	100,941,015	61,967,455	60,634	95.1	43,473,150 (70%)	43,001,188 (69%)	43,063,370 (69%)
GridION	GridION	SQK-LSK110	10,371,632	7,521,667	7360	96.0	5,461,630 (73%)	5,430,433 (72%)	5,462,816 (73%)
Q20	GridION	SQK-Q20EA	4,479,225	3,423,062	3349	97.9	2,501,260 (73%)	2,484,366 (73%)	2,511,016 (73%)
scmixology2 (Tian et al. [15])	PromethION	SQK-LSK109	36,927,546	25,164,948	101,471	95.7	18,347,232 (73%)	18,099,553 (72%)	17,818,239 (71%)

^a^Pass reads divided by the number of Cell Ranger barcodes

^b^Pass reads that have a valid 10x barcode and a UMI and can be assigned to a barcode in the whitelist

### Experimental workflow to assess the performance of BLAZE

We tested the performance of BLAZE by carrying out matched short- and long-read scRNA-seq on ~ 1000 human induced pluripotent stem cell (iPSC)-derived neural progenitors undergoing differentiation to the cortical lineage (Fig. 1B, see the “Materials and methods” section). Short reads were sequenced on an Illumina NOVA-seq to a high median depth of 96,000 reads per cell. Short-read data were analyzed with the Cell Ranger pipeline (10x Genomics) to generate a barcode whitelist that can be directly compared to a whitelist generated from long reads only. We performed long-read scRNA-seq using the FLT-seq protocol [15] and sequenced the sample on a PromethION flowcell generating ~ 62 million pass reads (Table 1). In addition to deep PromethION sequencing, we also sequenced the cDNA on the GridION using standard and higher-accuracy (Q20) chemistries generating ~ 7.5 and ~ 3.5 million pass reads, respectively (Table 1). This enabled us to assess the effects of read depth and variation in read accuracy on the performance of BLAZE, which is discussed in greater detail below. We also compared BLAZE to Sockeye (https://github.com/nanoporetech/Sockeye), the recently released ONT software for long-read scRNA-seq analysis that also generates a cell barcode whitelist from nanopore long-reads.

### BLAZE identifies high-confidence cell barcodes

Maximizing sequencing depth per cell is key to accurately identifying and quantifying isoforms in single-cell data [3]. Therefore, we first compared the performance of BLAZE to Sockeye and short-read barcodes detected by Cell Ranger in the higher-depth PromethION dataset. Cell Ranger (from short-read scRNA-seq), BLAZE, and Sockeye (from long-read scRNAs-seq) identified 1022, 804, and 1518 cell barcodes, respectively (Table 2). A comparison of barcodes showed 99.4% of barcodes identified by BLAZE were also found by Cell Ranger and Sockeye. However, a significant proportion of barcodes were unique to Cell Ranger and Sockeye (Fig. 1C). Analysis of cell barcode rank plots revealed BLAZE cell-associated barcodes had high read support in all methods (Fig. 1D). In contrast, unique Cell Ranger barcodes were often supported by few long reads, regardless of the different strategies of counting barcodes in BLAZE and Sockeye, suggesting that some barcodes identified from short-read sequencing were not well represented in the long-read dataset (Fig. 1D). Similarly, many unique Sockeye barcodes had little or no short-read support, suggesting they are unlikely to be associated with cells. Further supporting this possibility, BLAZE counts for unique Sockeye barcodes were much lower (median 4.5-fold) than for barcodes found by both methods, suggesting many of the long reads supporting unique Sockeye barcodes were low quality and the barcodes potentially false positives.

**Table 2:** Number of barcodes detected

Dataset ID	Cell Ranger^a^	BLAZE	Sockeye	BLAZE high sensitivity
PromethION	1022	804	1518	1033
GridION	1022	802	1016	1016
Q20	1022	804	1015	1022
scmixology2 (Tian et al. [15])	248	188	522	270

^a^From matched short-read data

The cell barcodes identified by Cell Ranger, BLAZE, and Sockeye enabled the downstream analysis of a very similar proportion of reads, 70%, 69%, and 69%, respectively (useable reads, Table 1), demonstrating that the smaller number of barcodes found by BLAZE does not negatively affect the overall proportion of reads that can be assigned to a cell. Together, these results show BLAZE provides an accurate list of long-read cell barcodes with little loss of sensitivity.

### Cell Ranger and Sockeye identify barcodes that are poorly supported by long reads

**Fig. 2:**
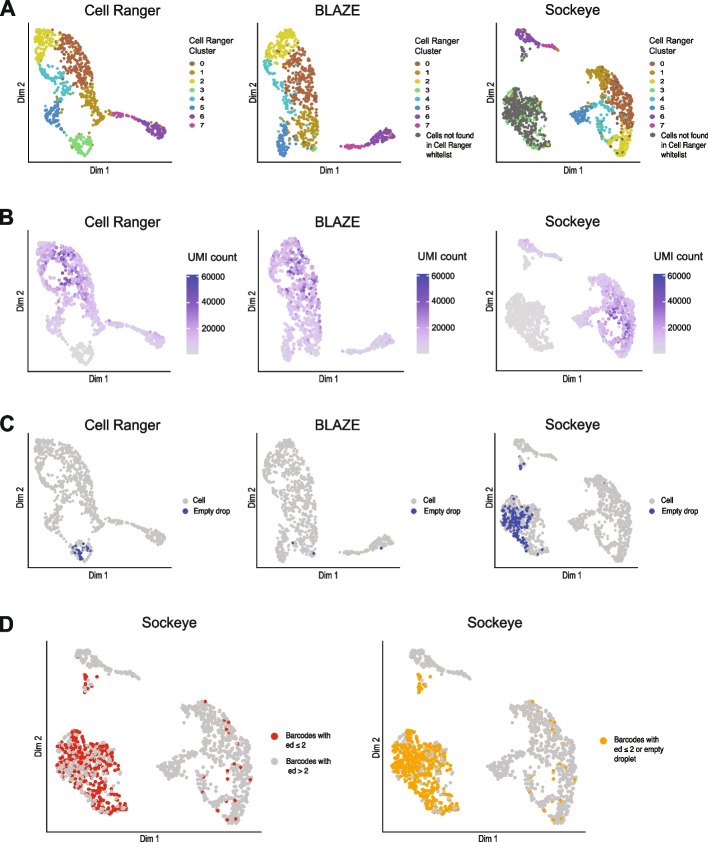
Comparison of cell clusters identified with BLAZE, Cell Ranger, and Sockeye barcodes. Isoform expression UMAP plots from PromethION data. Isoform counts were generated with FLAMES using barcode whitelists from either Cell Ranger, BLAZE, or Sockeye. **A** Cells in all three plots are colored based on clustering with the Cell Ranger whitelist. Cells not found in the Cell Ranger whitelist are colored in gray.** B** Cells colored based on UMI counts (sum of all unique UMIs across all transcripts) per cell. **C** Cells that are empty droplets colored in blue. **D** Sockeye UMAP colored based on edit distance ≤ 2 or empty droplet

We next asked if the barcode whitelists produced by Cell Ranger, BLAZE, and Sockeye would yield similar results when clustering cells based on gene or isoform expression. We used the barcode whitelists and the ~ 62 million long-reads from the PromethION as input into FLAMES [15] to produce gene and isoform counts and then generated UMAP plots in Seurat [20]. To facilitate a comparison between the methods, we made each UMAP plot separately and then colored each cell according to its assigned cluster using the Cell Ranger whitelist. This revealed both Cell Ranger and Sockeye identify an additional cluster not found by BLAZE. This result was consistent for analyses using either isoform (Fig. 2A) or gene (Additional file 1: Fig. S2A) counts and was further confirmed by re-coloring the cells based on the BLAZE clusters (Additional file 1: Fig. S2B). This cluster contained poorly supported barcodes, as demonstrated by the low UMI counts and low numbers of genes and isoforms detected in each “cell” (Fig. 2B and Additional file 1: Fig. S2C, D).

### Additional cell cluster found with Sockeye and Cell Ranger barcodes corresponds to empty droplets and barcodes with sequencing errors

We sought to determine why both Cell Ranger and Sockeye identified an additional cluster of “cells” not found by BLAZE. We first asked whether any barcodes likely represented empty droplets instead of actual cells. We performed an empty drops analysis using the R package DropletUtils [21] to identify cells that contained an expression profile not significantly different from the ambient RNA signature. The Sockeye, Cell Ranger, and BLAZE whitelists identified 211, 37, and 3 empty droplets, respectively. These Sockeye and Cell Ranger empty droplets were specific to the additional cluster not found in BLAZE (Fig. 2C).

In the case of the Cell Ranger whitelist, 36% of the additional cluster not found by BLAZE (cluster 3) are classified as empty droplets. These empty droplets and the remaining “cells” in cluster 3 likely represent those barcodes that exist in the matched short-read dataset but are poorly represented in the long-read data. Due to their low UMI counts, comparatively low number of genes and isoforms, and commonly ambient RNA signatures, these “cells” would be uninformative in a long-read scRNA-seq analysis.

The additional cluster found using the Sockeye whitelist contained 51% empty droplets (Fig. 2C, D). We next asked if many of the remaining barcodes in this cluster are likely false positives generated from nanopore sequencing errors, as Sockeye has no process to filter these out. We hypothesized that false-positive barcodes from sequencing errors would be more similar in sequence to existing barcodes than the random selection of 10x barcodes real cells would represent. To test this, we compared the edit distance of Sockeye-only barcodes to the Cell Ranger barcode whitelist and found that 60% of the Sockeye-only barcodes have an edit distance (ED) of ≤ 2 from real barcodes, which is significantly higher (*p* < 2.2E − 16, 1-sample proportion test) than the ~ 1% expected for a random set of 10x barcodes. These barcodes were highly enriched in the additional sockeye cluster not found by BLAZE (Fig. 2D), confirming that Sockeye only barcodes are often derived from sequencing errors.

Taken together, these analyses confirmed that the additional barcodes in both Cell Ranger and Sockeye often correspond to empty droplets instead of cells (in the case of Cell Ranger and Sockeye) and/or barcodes derived from sequencing errors (in the case of Sockeye). We note that BLAZE does not identify all Cell Ranger barcodes, and we theorize that some of these additional cells are likely recovered by Cell Ranger using an inbuilt empty drops algorithm, which identifies cells below the barcode threshold that have an expression profile which differs significantly from the ambient profile. BLAZE is unable to call these cells by default as gene and isoform expression quantification is needed to determine the ambient RNA signature. However, BLAZE can output the files necessary to perform such an analysis and recover additional barcodes (see the “Increasing barcode recall with BLAZE” section).

### Clustering based on the BLAZE whitelist separates biologically distinct cell types

**Fig. 3:**
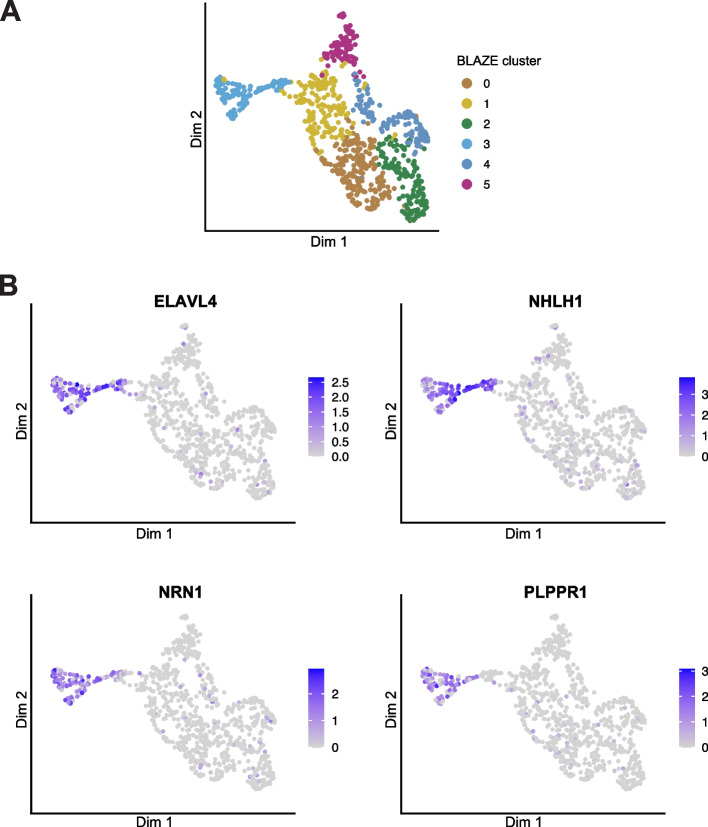
Gene expression UMAP colored by cluster and expression of marker genes. **A** UMAP showing clustering based on gene counts generated from FLAMES using the BLAZE whitelist. **B** UMAP colored by the expression of 4 marker genes known to be associated with differentiation and neuron development. The expression scale is colored based on Seurat normalized counts. Color scales are not comparable between the plots

The ~ 1000 cells analyzed here are in the early stages of cortical neuron differentiation; hence, it was important to confirm BLAZE whitelist-based cell clustering was due to distinct biological profiles and not as a result of sequencing depth per cell or non-biologically relevant factors. Marker gene analysis confirmed biologically meaningful gene expression differences between clusters (Additional file 2: Table S1 and Additional file 3: Table S2). We identified significant differences in the expression of hundreds of genes including key transcription factors such as *ELAVL4* and *NHLH1* (Fig. 3), which are known to be upregulated during the differentiation of cortical neurons [22, 23]. Moreover, we find differential gene expression of well-defined neuron-specific genes such as *NRN1* [24] and *PLPPR1* [25] (Fig. 3). In contrast, marker gene analysis on the additional clusters found using Cell Ranger and Sockeye barcodes found 7 and 8 DE genes, respectively (Additional file 4: Tables S3, S4). No biological relevance could be assigned to these expression changes, in line with these clusters largely representing empty droplets and non-cell associated barcodes. Together, these findings confirm that BLAZE cell clusters are transcriptionally distinct and that the BLAZE-FLAMES long-read pipeline is capturing the biological signal of neuronal differentiation.

While the use of FLAMES for isoform identification and quantification enables a fair comparison between whitelists, we wanted to ensure the false-positive detections from Sockeye were not a result of the FLAMES pipeline. To address this possibility, we implemented the complete Sockeye pipeline using default parameters and interrogated the UMAP plots generated by Sockeye. The Sockeye pipeline retained the additional cluster with low UMI counts (Additional file 1: Fig. S3A). We also note that Sockeye is currently limited to performing gene-based analyses and does not perform the isoform-based analyses enabled by long-read scRNA-seq. Overall, we find the BLAZE whitelist enabled the most accurate downstream expression and cell-type clustering of long-read scRNA-seq data.

### Barcode detection with BLAZE is robust to changes in read depth or read accuracy

We investigated the impact of read depth and sequencing accuracy on the results of BLAZE. We sequenced the same single-cell cDNA sample on the lower-output Nanopore GridION, using both the LSK110 and higher accuracy Q20 chemistries. We find that although the LSK110 and Q20 GridION data produce significantly fewer total and pass reads compared to the PromethION (approximately 10% and 5%, respectively) (Table 1), the number of barcodes found by BLAZE is virtually unchanged (Table 2). The Q20 GridION data is both lower depth and higher accuracy than the LSK110 data, leading to the possibility that higher read accuracy (via an increased proportion of high-confidence barcodes) could be maintaining barcode numbers. However, downsampling the LSK110 GridION data to match the Q20 read depth returned the same number of barcodes (802). We also downsampled the Q20 data to an average of 500 reads per cell to test the performance of BLAZE when there are very low numbers of reads per cell. BLAZE returned an almost identical 812 barcodes, demonstrating that it performs consistently across datasets with variable read depths and different sequencing accuracies. In addition, we observed a similar proportion of usable reads between datasets (Table 1), implying that the improved Q20 accuracy had minimal effect on the number of reads that can be assigned to a cell.

**Fig. 4:**
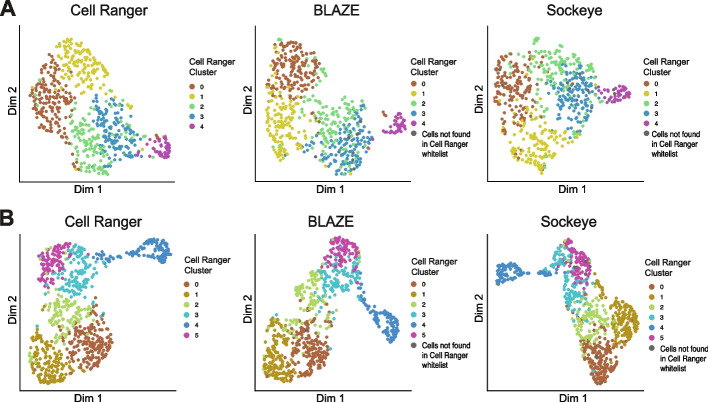
Isoform expression UMAP plot from Q20 and GridION data.** A** Q20. **B** GridION LSK110. Isoform counts were generated with FLAMES using barcode whitelists from either Cell Ranger, BLAZE, or Sockeye. Cells are colored as per Fig. 2A

We also assessed if Sockeye performed consistently across datasets of varying read depths. Sockeye identified 1016 and 1015 barcodes for LSK110 GridION and Q20 datasets, respectively (Table 2 and Additional file 1: Fig. S4), which was a significant reduction on the 1518 barcodes from the PromethION data. UMAP results based on FLAMES quantification for the lower-depth LSK110 and Q20 datasets revealed similar clustering between the methods (Fig. 4). The number of barcodes detected by Sockeye (and subsequent downstream results) is therefore heavily dependent on per-cell read depth, leading to inconsistent results, with a worse performance at higher read depths where cell-type separation and isoform profiling is enhanced.

We again tested if the full Sockeye pipeline would provide improved results over using the Sockeye barcodes in FLAMES. In contrast, we find that irrespective of the sequencing library used, quantification and UMAP generation using the Sockeye pipeline cluster cells in large part based on total UMI counts (Additional file 1: Fig. S3). A UMI-associated clustering effect could potentially represent a real biological signal if it related to cells undergoing differentiation and changing their transcriptional activity. However, using the BLAZE-FLAMES-Seurat pipeline (instead of the complete Sockeye pipeline), we do not see such strong correlations between clusters and UMIs (Additional file 1: Fig. S5). These findings confirm the Sockeye pipeline is impacted by UMI-associated confounders which bias UMAP results.

### BLAZE correctly identifies barcodes in long-read single-cell data of known cell lines

**Fig. 5:**
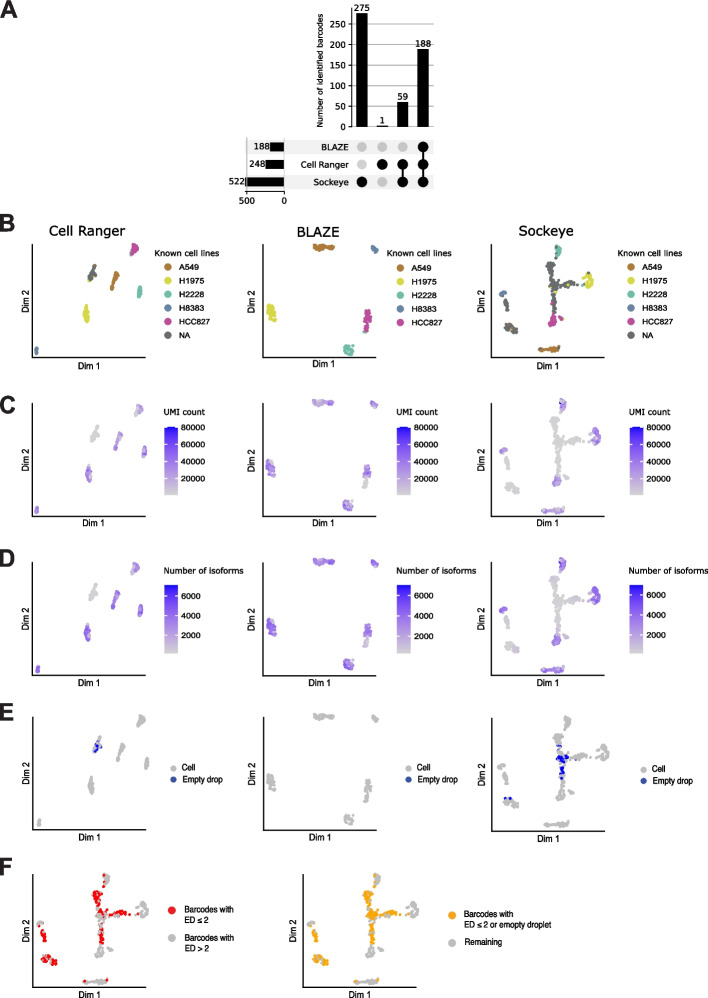
Barcode identification and clustering of Scmixology2 data.** A** Barcode upset plot comparing different whitelists. The bar chart on left shows the total number of barcodes found by each tool. Bar chart on top shows the number of barcodes in the intersection of whitelists from specific combinations of methods. The dots and lines underneath show the combinations.** B**–**D** Isoform expression UMAP plots: Isoform counts were generated with FLAMES using a barcode whitelist from either Cell Ranger (left), BLAZE (middle), or Sockeye (right). Cells are colored based on known cell types from Tian et al. [15] (**B**), total UMIs per cell (**C**), number of isoforms detected in each cell (**D**), and cells that are empty droplets (**E**). **F** Sockeye UMAP colored based on edit distance ≤ 2 or empty droplet

To further validate the performance of BLAZE, we compared Cell Ranger, BLAZE, and Sockeye on an additional long-read single-cell dataset containing known and distinct cell lines. We utilized the scmixology2 data from Tian et al. [15], which contains equal mixes of five cancer cell lines (~ 40 cells per line) profiled with matched Illumina and Nanopore reads. Cell Ranger (from the matched short-reads), BLAZE, and Sockeye identified 248, 188, and 522 cell barcodes, respectively (Table 2). Similar to the cortical differentiation dataset, we find all barcodes identified by BLAZE were also found by Cell Ranger and Sockeye (Fig. 5A). There were 59 barcodes identified by Cell Ranger and Sockeye but not by BLAZE and 275 barcodes unique to Sockeye (Fig. 5A). The larger number of cell barcodes identified by Sockeye compared to the number of cells sequenced further suggested it is identifying non-cell associated barcodes.

Implementation of the FLAMES pipeline for gene and isoform quantification supported the accurate identification of barcodes by BLAZE and confirmed the presence of Cell Ranger and Sockeye barcodes that did not reflect genuine cells in the long-read data (Fig. 5B–E). scmixology2 contained five distinct cell lines, and Tian et al. identified the barcodes belonging to each cell line in the long-read data (see Tian et al. [15] for details). We overlaid this information onto the UMAP plots generated from long reads (Fig. 5B). UMAP plots generated from BLAZE barcodes detected the five expected cell lines. All cells found by BLAZE were present in the matched short-read data (Fig. 5A), supporting the assertion that BLAZE accurately identifies cell barcodes while minimizing false-positive detections. In contrast, Cell Ranger barcodes identified six distinct clusters. Five corresponded to the cancer cell lines in this sample (Fig. 5B), while the sixth cluster (denoted as N.A) largely comprised barcodes with no cell line match. All these barcodes had very low cellular UMI counts and few unique isoforms (Fig. 5C, D), and 23 were identified as empty droplets (Fig. 5E) and therefore likely represent cells present in the short-read data but with few/no reads in the long-read data.

Clustering based on the Sockeye whitelist identified multiple additional cell-type clusters, with the majority (52%) of barcodes in clusters not matching one of the known cell lines. These “cells” all have low UMI counts and fewer detected isoforms (Fig. 5C, D), 47 have a background expression profile and are classed as empty droplets, and 135 are likely derived from sequencing errors present in 16-bp barcode sequence (Fig. 5E, F), highlighting that these barcodes are unlikely to represent real cells. To ensure these findings were not a consequence of the FLAMES pipeline, we also ran the entire Sockeye workflow. The Sockeye-generated UMAP displayed similar results (Additional file 1: Fig. S6), further supporting incorrect barcode identification by Sockeye. The identification of empty droplets and spurious cell clusters when using the Cell Ranger and Sockeye whitelists again demonstrates that BLAZE produces a more accurate representation of barcodes present in long-read datasets.

### Increasing barcode recall with BLAZE

**Fig. 6:**
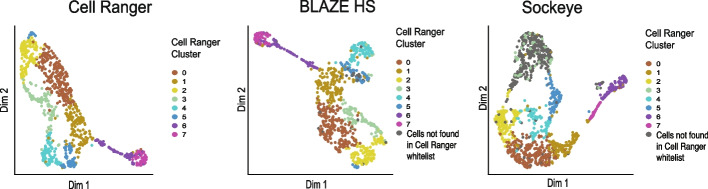
UMAP plots from PromethION data with BLAZE high sensitivity (HS) mode. Counts were generated with FLAMES using barcode whitelists from either Cell Ranger, Sockeye, or BLAZE HS. Isoform expression UMAP colored by Cell Ranger clusters: Empty droplets were removed prior to clustering

Some long-read single-cell analyses may prioritize high recall of the barcodes present. To enable this, we implemented two features in BLAZE: firstly, a high sensitivity (HS) mode, which aims to identify a larger set of possible barcodes present in the long-read data; secondly, an option to output a list of known background barcodes with an ED > 4, which can be used in an empty drops analysis to identify cells with ambient RNA expression and/or recover additional cells that are below the detection threshold. BLAZE HS mode identifies 1033 barcodes in our PromethION data and 270 barcodes in the scmixology2 dataset, with a higher level of overlap between the Cell Ranger and BLAZE HS whitelists (Additional file 1: Fig. S7A). However, BLAZE HS identifies the additional cluster found by Cell Ranger and identifies barcodes not found in the Cell Ranger whitelist (Additional file 1: Fig. S7B). BLAZE HS mode trades higher recall (i.e., more true barcodes) for potentially lower precision (i.e., more non-cell associated barcodes), and therefore, we recommended running an empty drops analysis and removing cells with an ambient profile if using BLAZE HS mode. Using this combined methodology, we demonstrate a high level of concordance between Cell Ranger and BLAZE HS, while Sockeye identifies many non-cell-associated barcodes even after the removal of empty droplets (Fig. 6).

### Overall comparison between BLAZE and Sockeye

**Fig. 7:**
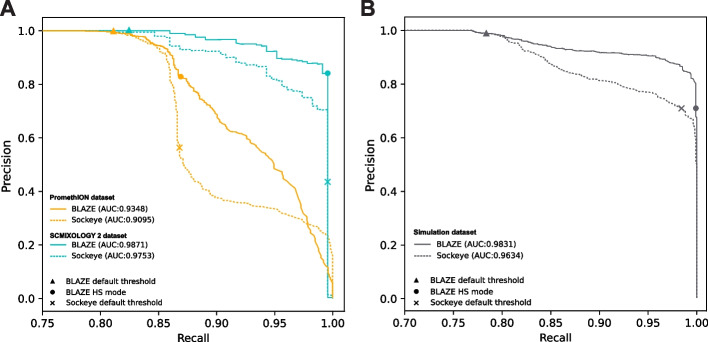
Precision-recall curves across** A** real and** B** simulated datasets for BLAZE and Sockeye. Precision and recall were calculated across different count thresholds by defining the barcodes identified from short reads as the ground truth, specifically the whitelist from Cell Ranger after the removal of empty droplets (**A**) and data simulated to the Cell Ranger whitelist to make it a perfect ground truth (**B**). The numbers in the legend show the area under the curve (AUC) values

BLAZE is more conservative than Sockeye in calling barcodes and therefore minimizes false-positive detections. However, both BLAZE and Sockeye use barcodes with counts above a threshold to generate the whitelist. Users of BLAZE have the flexibility to choose the count threshold in addition to using the default and HS modes. We therefore asked if BLAZE outperforms Sockeye generally. Using all four datasets above (Tables 1 and 2) and defining the cell barcodes identified by Cell Ranger minus barcodes identified as empty droplets as the ground truth, we calculated precision-recall curves across different count thresholds in BLAZE and Sockeye. The results demonstrated that BLAZE consistently outperforms Sockeye (Fig. 7A, Additional file 1: Fig. S8A) and outputs a better whitelist regardless of whether users prefer high precision or recall.

Using real data, it is not possible to generate a perfect ground truth barcode whitelist, even after removing identifiable empty droplets from the Cell Ranger whitelist. Therefore, we created a software pipeline SLSim (https://github.com/youyupei/SLSim) to simulate nanopore single-cell long reads, allowing us to compare BLAZE and Sockeye with a known ground truth barcode whitelist. Precision-recall curves across different count thresholds confirmed BLAZE consistently outperforms Sockeye (Fig. 7B, Additional file 1: Fig. S8B).

BLAZE is easy to install and run (see Additional file 5: Tables S5 for the runtime of BLAZE). However, a fair runtime comparison between BLAZE and Sockeye is difficult because Sockeye is not designed to solely generate a barcode whitelist but instead runs the whole pipeline for single-cell gene expression and therefore requires a longer runtime. Sockeye also needs to perform mapping to identify putative barcodes while BLAZE does not. In addition, Sockeye cannot be utilized as a stand-alone tool to perform single-cell isoform analysis (for which long reads are significantly more useful than short reads) as it only performs gene-level quantification. In this sense, running BLAZE is quicker, and the integration is easier as BLAZE outputs a whitelist using the Cell Ranger format that can be input into tools such as FLAMES without modification.

## Discussion

Single-cell RNA sequencing (scRNA-seq) has revolutionized the study of transcriptomes, yet it is limited by the use of short-read sequencing methods. With recent advancements in long-read scRNA-seq methodologies [5, 26] and analysis tools [27], the potential to study the complete array of RNA isoforms and quantify isoform expression at single-cell resolution is becoming possible. The use of “noisy” long reads, however, presents its own unique set of challenges, primarily the difficulty in identifying the cell barcodes needed to assign each transcript to its cell of origin. Consequently, the use of matched short-read data has been fundamental to the successful implementation of high-depth, high-throughput nanopore long-read scRNA-seq. In spite of the higher error rate of nanopore reads, we show that BLAZE aids in eliminating the need for matched short-read sequencing. This not only simplifies the procedure but also reduces overall library construction and sequencing costs and therefore increases the accessibility of long-read scRNA-seq.

We found BLAZE to be robust in its ability to accurately identify 10x cell barcodes from long reads. BLAZE can be applied to different types of single-cell samples and performs equally well on both higher accuracy Q20 data, as well as lower accuracy reads generated from ONT’s LSK110 and LSK109 protocols. We find that ONT’s recently published software for long read-only barcode identification, Sockeye, appears to be affected by read-depth associated confounders and identifies false-positive cell barcodes that are derived from sequencing errors. An alternate possibility is that Sockeye is more effective than BLAZE at identifying cell barcodes and therefore finds larger numbers of cells. However, this seems unlikely given Sockeye finds much larger numbers of long-read barcodes than matched short-read sequencing, unique Sockeye barcodes do not match the known cell types present in the scmixology2 data, and the unique “cells” often represent empty droplets and have very low numbers of UMIs, genes, and unique isoforms. As a consequence of the BLAZE software and pre-print becoming publicly available prior to publication, Sockeye has been depreciated and BLAZE’s qscore filtering step and quantile-based approach incorporated into ONT’s replacement software pipeline wf-single-cell.

In order to accurately identify and quantify isoforms from scRNA-seq, it is important to sequence cells deeply [3]. BLAZE showed the greatest advantage over other methods in the higher-depth PromethION datasets and therefore performs well in the context most relevant to long-read scRNA-seq. At the same time, the performance of BLAZE is largely independent of read depth. BLAZE produced consistent results in datasets with > 60 k reads/cell down to only ~ 500 reads/cell, demonstrating it is applicable to a wide variety of samples. We designed BLAZE to be simple to install and use and seamlessly integrate into existing isoform identification and quantification pipelines such as FLAMES [15], meaning no modifications to the existing protocols or pipelines are needed. This provides a further advantage over Sockeye, which currently only performs gene-level quantification. Importantly, we find that BLAZE performs comparably well, if not better, than Cell Ranger whitelists generated from matched short-read data. More than 99% of barcodes identified with BLAZE were present in the short-read whitelist confirming that false-positive detections with BLAZE are rare. Conversely, > 20% of barcodes identified by Cell Ranger were not found by BLAZE default mode. These barcodes were supported by few long reads, expressed comparatively few genes and isoforms, and often had an ambient RNA profile. We hypothesize that despite sequencing matched samples, some cells (and hence barcodes) found in short-read data are poorly represented among the long reads. We theorize that the separate PCR reactions in the long- and short-read library preps, either by chance and/or bias, led to some cells dropping out of the libraries. Supporting this, the Cell Ranger knee plot showed that barcodes not found by BLAZE had low UMI counts in the short-read data. Such barcodes are the most likely not to be found in matched long-read sequencing due to chance, biases, or differences in read depths. Consequently, the use of long read-only barcode identification methods should produce whitelists that more faithfully represent cells profiled with long-read sequencing.

The accurate identification of single-cell barcodes is crucial to downstream gene and isoform quantification. Nearly all single-cell workflows cluster cells based on expression using dimensional reduction techniques such as t-SNE [28] and UMAP [29, 30]. These methods enable further integration of cell type-specific markers and can be used to identify differentially expressed genes and isoforms between cell clusters. Spurious cells often cluster together, giving a misleading impression of additional cell clusters, which could confound differential expression analyses and biological interpretation of the results. Furthermore, usable reads are assigned to non-cell associated barcodes, reducing the read depth of real cells and decreasing experimental power for isoform identification and quantification. We find around 7% of pass reads are assigned to non-cell associated Sockeye barcodes. Filtering out cells that have low UMI counts can reduce false-positive cells; however, deciding on an appropriate UMI filtering threshold can be difficult and would depend on sequencing read depth and the transcriptional activity of the cells. It can be challenging to distinguish between cells that produce small amounts of RNA (and subsequently have few UMIs) and non-cell-associated barcodes, while some non-cell-associated barcodes can be assigned enough reads to have an expression profile largely indistinguishable from real cells. Tools designed to generate single-cell barcode whitelists should therefore prioritize high precision as false-positive barcodes can confound downstream workflows.

While long-read scRNA-seq is becoming more feasible as sequencing outputs increase and analysis packages are designed, not all analysis challenges are completely solved. Along with barcode identification, the identification and collapse of UMIs, assignment of reads to identified barcodes, and construction of accurate isoforms are all areas of active research. Further progress in these areas will be important to enable higher accuracy isoform identification, quantification, and differential expression analyses from long-read scRNA-seq data.

A limitation of the current study is the use of Cell Ranger as the ground truth to determine the precision-recall of BLAZE and Sockeye, since our results suggest some barcodes identified by Cell Ranger do not represent genuine cells in the long-read data. Our simulation data largely solves the issue of a ground truth; however, it still contains limitations with regard to correctly modeling nanopore read error distributions. Specifically, modeling an error distribution that accurately generates (1) a higher frequency of errors at the start and end of reads (in which the 10x barcode lies) and (2) a higher frequency of adjacent errors, as is the case for nanopore reads, is a challenge. These likely cause our simulated data to overstate the performance of Sockeye; even so, we find BLAZE precision-recall systematically outperforms Sockeye in both real and simulated data. We therefore conclude that the outperformance would be even greater with a ground truth dataset that perfectly reconstitutes the nanopore error profile.

Currently, BLAZE is limited to identifying 10x single-cell barcodes from nanopore reads. Although other long-read single-cell methodologies such as scCOLOR-seq [13] and R2C2 [31] have been used to profile single cells with long reads, the 10x chromium platform is the most widely available and popular platform. We therefore designed the initial version of BLAZE to facilitate 10x barcode identification. Recent developments in throughput and accuracy for PacBio HiFi sequencing are increasing the applicability of PacBio for long-read scRNA-seq, while long-read nanopore protocols for other scRNA-seq modalities such as Split-seq are also now available [14, 32–35]. Although BLAZE is currently limited to the identification of 10x barcodes from nanopore reads, we see the potential to expand BLAZE to process both PacBio HiFi reads and reads from other scRNA-seq methods in the future.

## Conclusion

We show that BLAZE is a highly accurate single-cell barcode identification tool for nanopore long reads. We demonstrate that BLAZE works well across different datasets, read depths, and read accuracies and can seamlessly integrate into existing tools for downstream gene and isoform identification and quantification. Crucially, BLAZE eliminates the requirement for additional matched short-read data and therefore simplifies long-read scRNA-seq protocols while significantly reducing cost. BLAZE has been designed to be widely accessible and easy to use and is available at https://github.com/shimlab/BLAZE.

## Materials and methods

### Cell lines and stem cell differentiation

RM3.5 human induced pluripotent stem cells (hiPSC) [36] were cultured under xenogeneic conditions in accordance with the protocol described in Niclis et al. [37]. RM3.5 cells (passages 18–20) were confirmed to be karyotypically normal, pluripotent (PluripotentTest, Thermo Fisher), and regularly tested for mycoplasma (MycoAlert kit, Lonza). PSCs were differentiated into cortical neuron lineage using the protocol described by Gantner et al. [38].

### Preparation of single-cell suspension

At day 26 post-neural induction, RM3.5 cells undergoing cortical differentiation were harvested for analysis. Cells were washed twice in 300 mL of DPBS − / − and exposed to Accutase (Innovative Cell Technologies, Inc., San Diego, CA, http://www.accutase.com) for 12 min at 37 °C. Following incubation, cells were moved to a 15-mL falcon tube and were gently triturated to help generate a single-cell suspension. DPBS was added at a 1:1 ratio to inactivate the Accutase and the sample gently centrifuged at 1500 rpm for 3 min at 4 °C and supernatant removed. Cells were resuspended in 2-mL DBPS and Rock inhibitor Y-27632 (diluted 1:1000) (Tocris Bioscience) to prevent cell death. The cell suspension was passed through a Flowmi™ strainer (Flowmi; Cat. No. 64709–60) to remove the remaining cell debris. Finally, cells were counted using a hemocytometer, and viability was assessed with trypan blue stain (Thermo Fisher Scientific Cat. No. 15250061) prior to the final resuspension in DPBS with 0.04% BSA and Rock inhibitor.

### FLT-seq 10x single-cell processing and cDNA amplification

FLT-seq was performed in accordance with the published protocol [15] (https://www.protocols.io/view/massively-parallel-long-read-sequencing-of-single-81wgbpp1nvpk/v1). Briefly, the cell suspension was prepared for the target recovery of 5000 cells, with 20% for matched short and long-read sequencing. Single-cell processing and cDNA amplification was performed in accordance with the 10x Genomics Chromium Single-cell 3′ gene expression protocol (v3.1), except that to generate full-length cDNA, the reverse transcription extension time was extended to 2 h. GEMs were split 80%:20%, with the cDNA from the 20% (~ 1000 cells) processed to create matched short- and long-read libraries. We performed 16 cycles for the short-read index PCR and 12 cycles for the long-read template generation. We used FLT-seq as this protocol generates a high proportion of full-length (3′ adaptor to 5′ TSO) reads and an almost negligible proportion of TSO artifacts (TSO-TSO reads without a valid cell barcode).

### Short-read Illumina sequencing

The Illumina short-read library was sequenced on the Novaseq6000 to a depth of 100 M reads. Basecalling and quality scoring were determined using Real-Time Analysis (RTA3) on board software, while the FASTQ file generation and de-multiplexing utilized bclConver v3.9.3.

### Nanopore single-cell library preparation and sequencing

Full-length cDNA generated from the FLT-seq protocol was prepared using the SQK-LSK110 Ligation Sequencing Kit (ONT) with the following modifications: incubation times for end-preparation and A-tailing were lengthened by 15 min, and all AMPureXP cleaning steps were performed at × 1.8. Libraries were sequenced on both the GridION (FLO-MIN106 flow cell) and PromethION (FLO-PRO002 flow cell) loading ~ 45 fmol with an additional flow cell top-up with any remaining library at 24 h. Fast5 files were generated using MinKnow v21.02.5 on the GridION and v22.03.4 on the PromethION and basecalled with guppy v6.0.7 with the super high-accuracy configuration file.

We prepared an additional long-read library with the SQK-Q20EA Genomic DNA by ligation Q20 + early access kit (ONT) with the same modifications stated above. We sequenced the Q20 library on the GridION (FLO-MIN112 flow cell), loading 10 fmol with an additional 10 fmol top-up at 24 h. Fast5 files were generated using MinKnow v21.05.25 and basecalled with guppy v6.0.7 with the dna_r10.4_e8.1_sup.cfg configuration file.

The median sequencing accuracy was calculated by first mapping pass FASTQ files to the transcriptome with Minimap2 [39] using the command minimap2 -ax map-ont $REF $FASTQ > trans_mapping.sam. The median accuracy was calculated using a custom R script found at https://github.com/josiegleeson/BamSlam [40]. In short, the cigar strings from primary alignments were extracted, and the total number of mismatches, insertions, and deletions per alignment was calculated.

### Identification of putative barcode sequence in each read

BLAZE identifies the likely position of the cell barcode (referred to as “putative barcode”) by first identifying the position of the adaptor. Similar to [9], in each nanopore read, BLAZE searches for the last 10-nt sequence of the adaptor (i.e., “CTTCCGATCT”) in the first 200 nt of the read. Specifically, BLAZE aligns the “CTTCCGATCT” to the first 200 nt of the read using Biopython [41] and allows up to 2 mismatches, insertions or deletions. This procedure ensures a high sensitivity in identifying the adaptor location but will potentially find multiple locations. Thus, BLAZE also requires a downstream polyT sequence for accurate identification of the adaptor location. Specifically, BLAZE conducts a lenient search that looks for 4 consecutive ‘T’s 20 ~ 50 nt downstream of the adaptor, as the polyT tail in nanopore reads is often truncated due to limitations in basecalling of homopolymers [12]. The corresponding adaptor is considered to be valid only if the polyT is found. BLAZE then repeats the same procedure for the reverse complement sequence. Reads with exactly 1 valid adaptor were kept for the downstream steps. The 16-nt sequence immediately downstream of the adaptor is defined as the “putative barcode.”

### Selection of high-quality putative barcodes

To accurately identify the sequences of barcodes, BLAZE selects high-quality putative barcodes that are less likely to contain basecalling errors. Basecalling outputs provide a (Phred) quality score for each base, which indicates the probability of the base being correctly basecalled. Incorrectly basecalled bases generally have a low quality score, so putative barcodes with error(s) are more likely to have at least one base with a low quality score. Therefore, for each putative barcode, BLAZE calculates the minimum quality score across the 16 bases in the putative barcode, denoted as “minQ,” and selects putative barcodes with minQ $\ge$ 15 as high-quality putative barcodes. See Additional file 1: Fig. S1 for our choice of 15 as a threshold.

### Identification of cell-associated barcodes from high-quality putative barcodes

BLAZE lists unique high-quality putative barcodes, counts their occurrences, and ranks them based on those counts. Next, similar to Zheng et al., BLAZE selects those barcodes whose counts are larger than a stringent count threshold $T$ as cell-associated barcodes (i.e., barcodes likely associated with cells) and outputs them in a whitelist. The threshold $T$ has been chosen as follows. For a given expected number of recovered cells, denoted by $N$, we obtain *c*, the count of a unique high-quality barcode whose rank is $0.95\times N$. Then, we use $0.05\times c$ as the threshold $T$. In practice, the targeted number of cells can be a plausible number for $N$. We use $N=500$ in the analysis in this manuscript. The number of barcodes in the final whitelist is robust to the choice of $N$ (Additional file 1: Fig. S9) as long as *N* is set within a reasonable range that is not too divergent from the true number (e.g., the number of barcodes change from 186 to 193 when *N* is increased from 50 to 1500 in the analysis of the scmixology2 dataset with ~ 200 cells). In the BLAZE HS mode that aims to identify a larger set of barcodes, we reduced the threshold to 10% of the standard threshold above.

### Barcode whitelist generation and gene and isoform qualification with FLAMES

We produced barcode whitelists using three software packages: Cell Ranger v6.0.2, Sockeye v0.2.1 (ONT) (https://github.com/nanoporetech/Sockeye), and BLAZE v1.1.0 (https://github.com/shimlab/BLAZE). First, we processed fastq files generated from the matched Illumina sequencing using the Cell Ranger pipeline to generate the barcode whitelist. Next, we ran the Sockeye pipeline and BLAZE on each long-read dataset using default parameters to generate barcode whitelists from long-reads only. We performed gene- and isoform-level qualification using FLAMES [15] (https://github.com/OliverVoogd/FLAMES) using an edit distance of 2, hg38 reference genome and GENCODE v31 comprehensive transcriptome. We used isoform count matrices generated by FLAMES to produce gene-level counts using a custom python script (available at https://github.com/youyupei/bc_whitelist_analysis/).

### UMAP generation and single-cell data processing

Gene and isoform count matrices were analyzed with the R package Seurat v4.1.1 [20]. We applied a minimum filtering threshold of 200 features (genes or isoforms) to remove cells with very low UMI counts in accordance with Seurat pipeline recommendations. Clustering was performed on all datasets with a resolution value of 0.7. Marker genes/isoforms that distinguish clusters were found using Seurat::FindMarkers using default parameters: the full workflow is available at https://github.com/youyupei/bc_whitelist_analysis/blob/main/script/SC_Marker_gene.Rmd. Seurat analysis scripts and output files can be found at https://github.com/youyupei/bc_whitelist_analysis.

### scmixology 2 dataset

Fast5 files from the scmixology 2 dataset published in Tian et al. [15] were rebasecalled with guppy v5.1.13 to generate fastq files. We generated long-read barcode whitelists using BLAZE and Sockeye as stated above. The Cell Ranger generated whitelist was obtained from matched Illumina short-read sequencing published in Tian et al. [15]. These three whitelists were inputs into FLAMES for gene and isoform quantification, and downstream processing with Seurat is as stated above. To determine the cell line for each barcode Tian et al. (2019) used Demuxlet55 [42], which uses the genetic variation between cell lines to identify the most probable identity of each barcode [43].

### Empty drops analysis and edit distance

To generate an ambient RNA expression profile to test for empty droplets, we randomly selected 5000 background barcodes with an edit distance > 4 from the Cell Ranger whitelist. We combined the background list of barcodes with the whitelists generated from Cell Ranger, BLAZE, and Sockeye producing three whitelists that we provided to FLAMES to generate gene and transcript count matrices. The large edit distance in the background barcode set prevents incorrect read assignment to barcodes that may be similar, meaning reads from true cells will not be assigned to background barcodes biasing the ambient RNA profile. To identify cell barcodes that show an ambient RNA expression profile, we used the R package DropletUtils version 1.12.2 [21] using default parameters and an FDR threshold of 1%.

We used a python package Levenshtien (https://pypi.org/project/python-Levenshtein/) to calculate the edit distance of Sockeye barcodes. We then extracted the set of Sockeye-only barcodes and a random set of 10x barcodes and compared the edit distance of both sets to the Cell Ranger whitelist. The full workflow is available at https://github.com/youyupei/bc_whitelist_analysis/blob/main/script/empty_drops_transcripts.Rmd.

### Generating simulation data

To simulate long-read data with the most realistic read count distribution, we first analyzed the short-read dataset and identified 360,279 barcodes using Cell Ranger, of which 1022 barcodes were identified to be cells. In our simulation, the 1022 barcodes were classed as true cells, and the remaining 359,257 barcodes were background so that the precision and recall of the barcode whitelist can be calculated. For each of the 360,279 barcodes, using the corresponding UMI count from short reads, we simulated the same number of long reads, so that the read count distributions of both true cells and background barcodes were identical to the short reads.

No existing simulation tool is specifically designed for single-cell long reads, and none of the existing long-read simulators provides options to add barcodes or UMIs. Therefore, we created a tool SLSim (https://github.com/youyupei/SLSim), which simulates single-cell long reads in two steps: (1) constructs an artificial error-free read (or “perfect read”) with a given barcode and UMI and (2) simulates errors into the perfect reads. A perfect read starts with a nanopore adaptor, followed by a 10x adaptor, cell barcode, UMI, 15-nt poly T, 200-nt mRNA fragment, and ends with a template switch oligo (TSO) sequence. The UMI was a randomly generated 12-nt sequence in each read, and the mRNA was randomly sampled from the GENCODE V31 transcript reference [44]. As both cDNA strands are sequenced in real experiments, each perfect read had an equal probability of either being converted to its reverse complement or kept in the original strand. Next, SLSim uses a python function from Badread [45] that can simulate nanopore errors and per-base quality scores based on its pre-trained models. We set the error distribution to globally replicate the error profile from our PromethION dataset. The script for running the simulation can be found at https://github.com/youyupei/bc_whitelist_analysis.

## Supplementary Information

**Additional file 1:Fig. S1.** Distribution of minQ. **Fig. S2.** UMAP plots from PromethION data. **Fig. S3.** Gene expression UMAP plot from Sockeye pipeline for PromethION, GridION and Q20 data. **Fig. S4.** Barcode upset plot comparing different whitelists. **Fig. S5.** Gene expression UMAP plot (using BLAZE whitelist) from PromethION, GridION and Q20 data. **Fig. S6.** Gene expression UMAP plot from Sockeye pipeline for scmixology 2 data. **Fig. S7.** Upset and UMAP plots for BLAZE HS mode without removing empty droplets. **Fig. S8.** Full range of precision-recall curves across real and simulated data. **Fig. S9.** Effect of specifying different numbers of expected cells in BLAZE.

**Additional file 2:Table S1.** Genes differentially expressed between BLAZE cell clusters.

**Additional file 3:Table S2.** Genes differentially expressed in the differentiating cell cluster (BLAZE analysis).

**Additional file 4:Table S3.** Genes differentially expressed in Cell Ranger cluster not found in BLAZE. **Table S4.** Genes differentially expressed in Sockeye clusters not found in BLAZE.

**Additional file 5:Table S5.** BLAZE runtime (with 32 and 6 cpus).

**Additional file 6.** Review history.

## Data Availability

Fast5 and fastq files are available from ENA under accession PRJEB54718 [46]. The processed data and scripts used in this study are available at https://github.com/youyupei/bc_whitelist_analysis/ [47]. BLAZE and SLSim are implemented in Python and available on GitHub at https://github.com/shimlab/BLAZE and https://github.com/youyupei/SLSim [48, 49] under GNU General Public License v3.0. The version of the software and analysis scripts used to produce the results in the paper have been deposited at https://doi.org/10.5281/zenodo.7700831 [50].
