# Supplementary material for: Identification of cell barcodes from long-read single-cell RNA-seq with BLAZE
Source: Genome Biol. 2023 Apr 6;24:66. doi: 10.1186/s13059-023-02907-y (PMC10077662; doi:10.1186/s13059-023-02907-y)
Supplement: Supplementary file 1 — Additional file 1:Fig. S1. Distribution of minQ. Fig. S2. UMAP plots from PromethION data. Fig. S3. Gene expression UMAP plot from Sockeye pipeline for PromethION, GridION and Q20 data. Fig. S4. Barcode upset plot comparing different whitelists. Fig. S5. Gene expression UMAP plot (using BLAZE whitelist) from PromethION, GridION and Q20 data. Fig. S6. Gene expression UMAP plot from Sockeye pipeline for scmixology 2 data. Fig. S7. Upset and UMAP plots for BLAZE HS mode without removing empty droplets. Fig. S8. Full range of precision-recall curves across real and simulated data. Fig. S9. Effect of specifying different numbers of expected cells in BLAZE. [file 13059_2023_2907_MOESM1_ESM.pdf]

Fig. S1

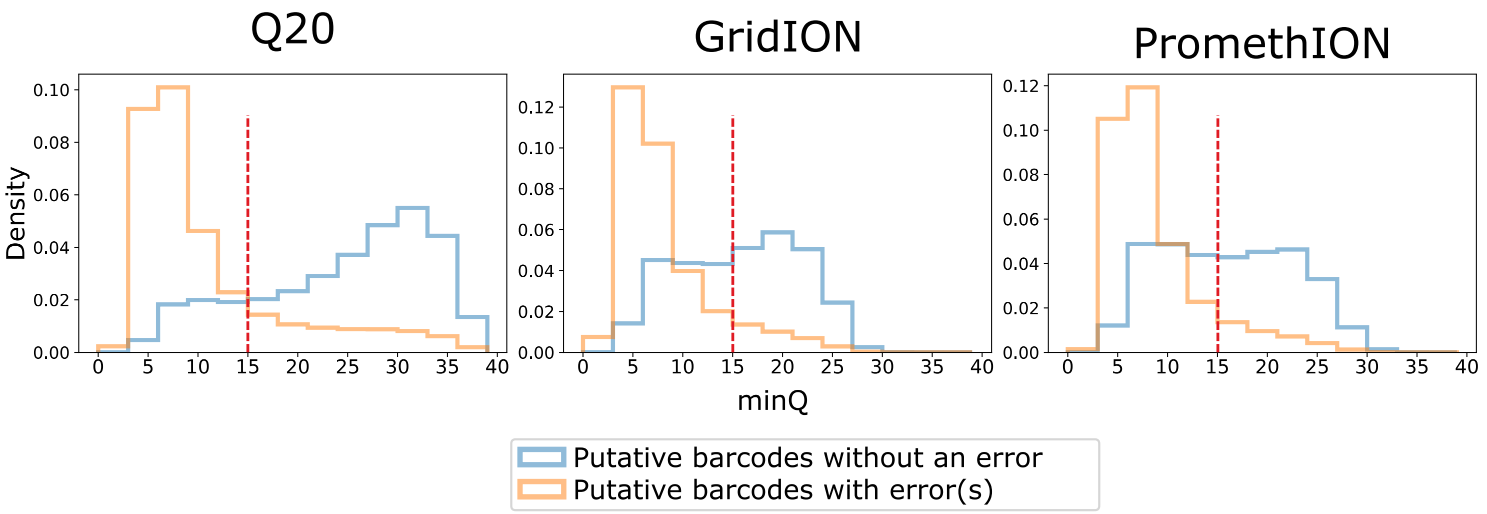

Fig. S1. Distribution of minQ: The title for each panel represents a different dataset in Table 1. Using the barcodes identified from short reads (by Cell Ranger) as a ground truth, all putative barcodes could be categorised as either: 1. Without error (exact match to ground truth barcode); 2. With error(s). While it's possible a putative barcode contains an error but exactly matches a ground truth barcode by chance, this should be very rare and not significantly impact the distribution. The red dotted lines indicates minQ=15, which is the threshold used in BLAZE to identify high-quality putative barcodes.

Fig. S2

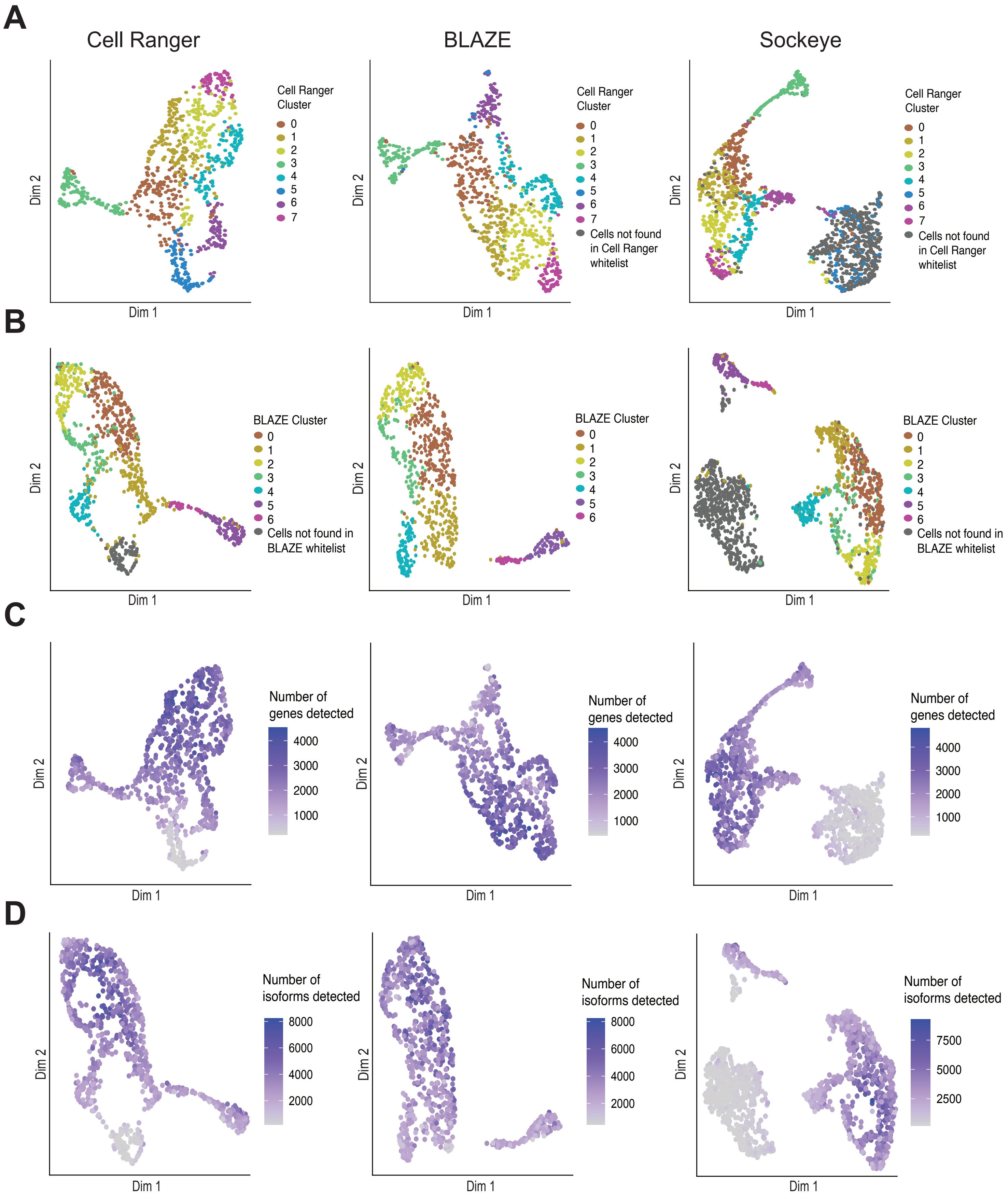

Fig. S2. UMAP plots from PromethION data. Counts were generated with FLAMES using barcode whitelists from either Cell Ranger, BLAZE or Sockeye. **A**. Gene expression UMAP coloured by Cell Ranger clusters: Cells in all three plots are coloured based on clustering with the Cell Ranger whitelist. Cells not found in Cell Ranger whitelist are coloured in gray. **B**. Isoform expression UMAP coloured by BLAZE clusters: Cells in all three plots are coloured based on clustering with the BLAZE whitelist. Cells not found in BLAZE whitelist are coloured in gray. **C**. Gene expression UMAP coloured by number of genes detected per cell. **D**. Isoform expression UMAP coloured by number of unique isoforms detected per cell: Unique isoforms are defined as structurally distinct RNA isoforms with a least one UMI count.

Fig. S3

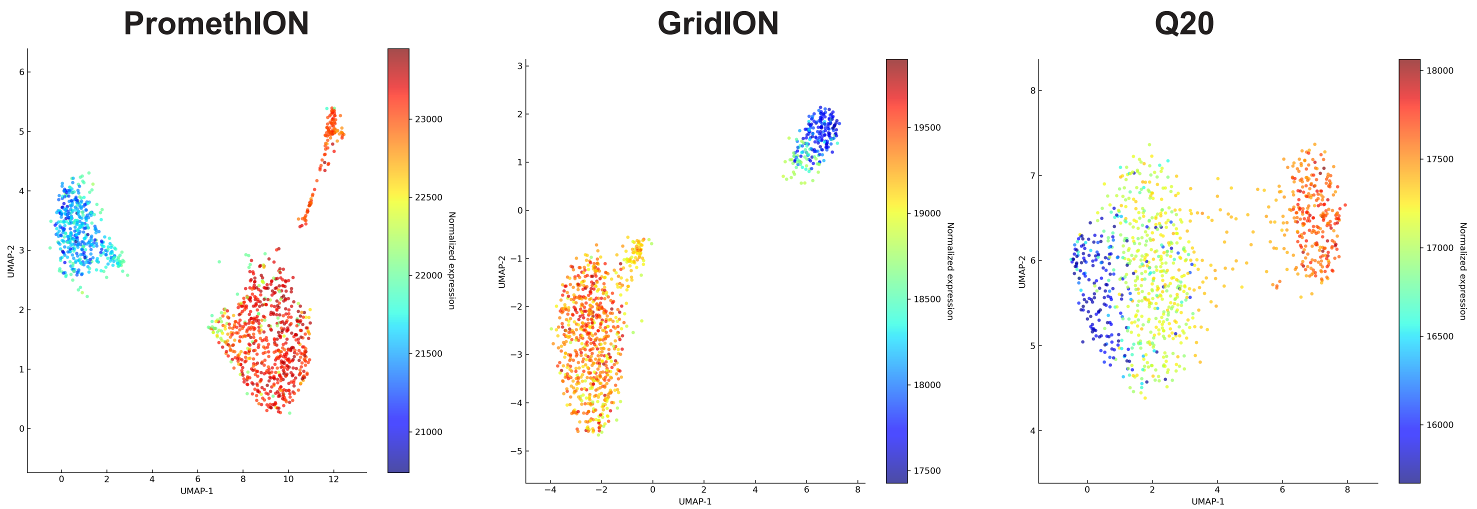

Fig. S3. Gene expression UMAP plot from Sockeye pipeline (PromethION, GridION and Q20 data). Cells are coloured based on normalised gene expression calculated within the Sockeye pipeline.

Fig. S4

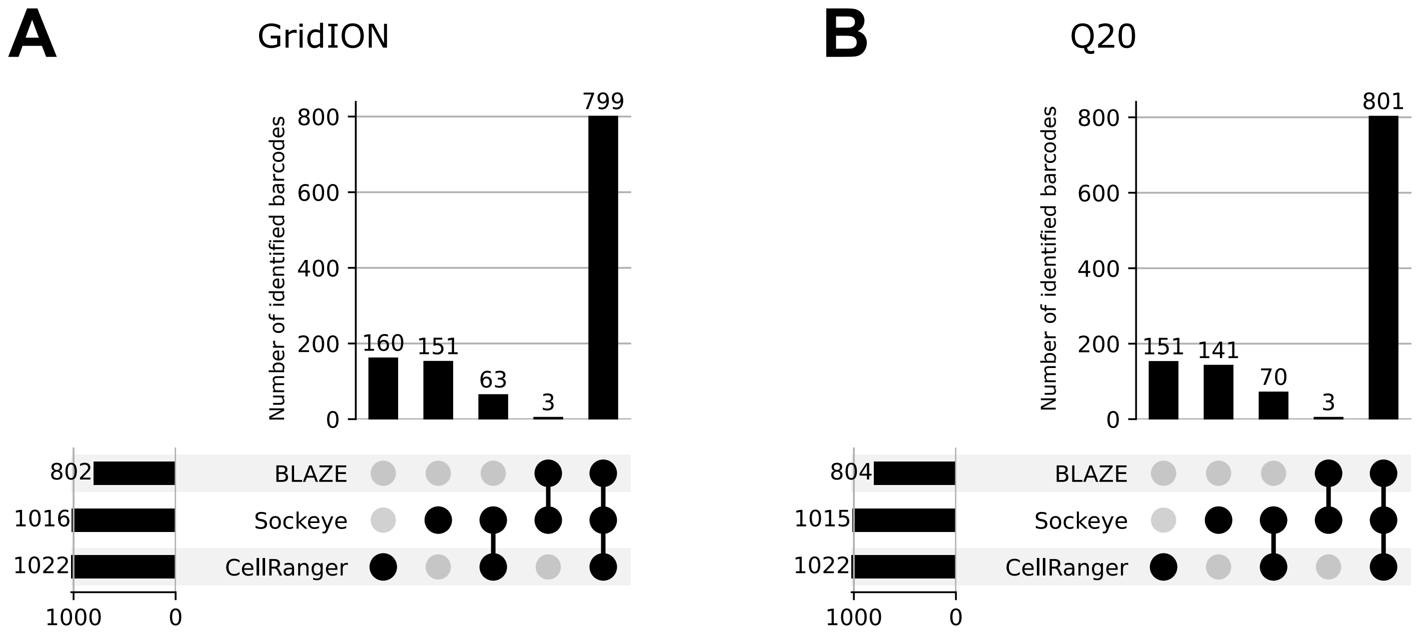

Fig. S4. Barcode upset plot comparing different whitelists in **A**. LSK110 GridION data and **B**. Q20 GridION data. The bar chart on the left shows the total number of barcodes found by each tool. The bar chart on the top shows the number of barcodes in the intersection of whitelists from specific combinations of methods. The dots and lines underneath show the combinations.

Fig. S5

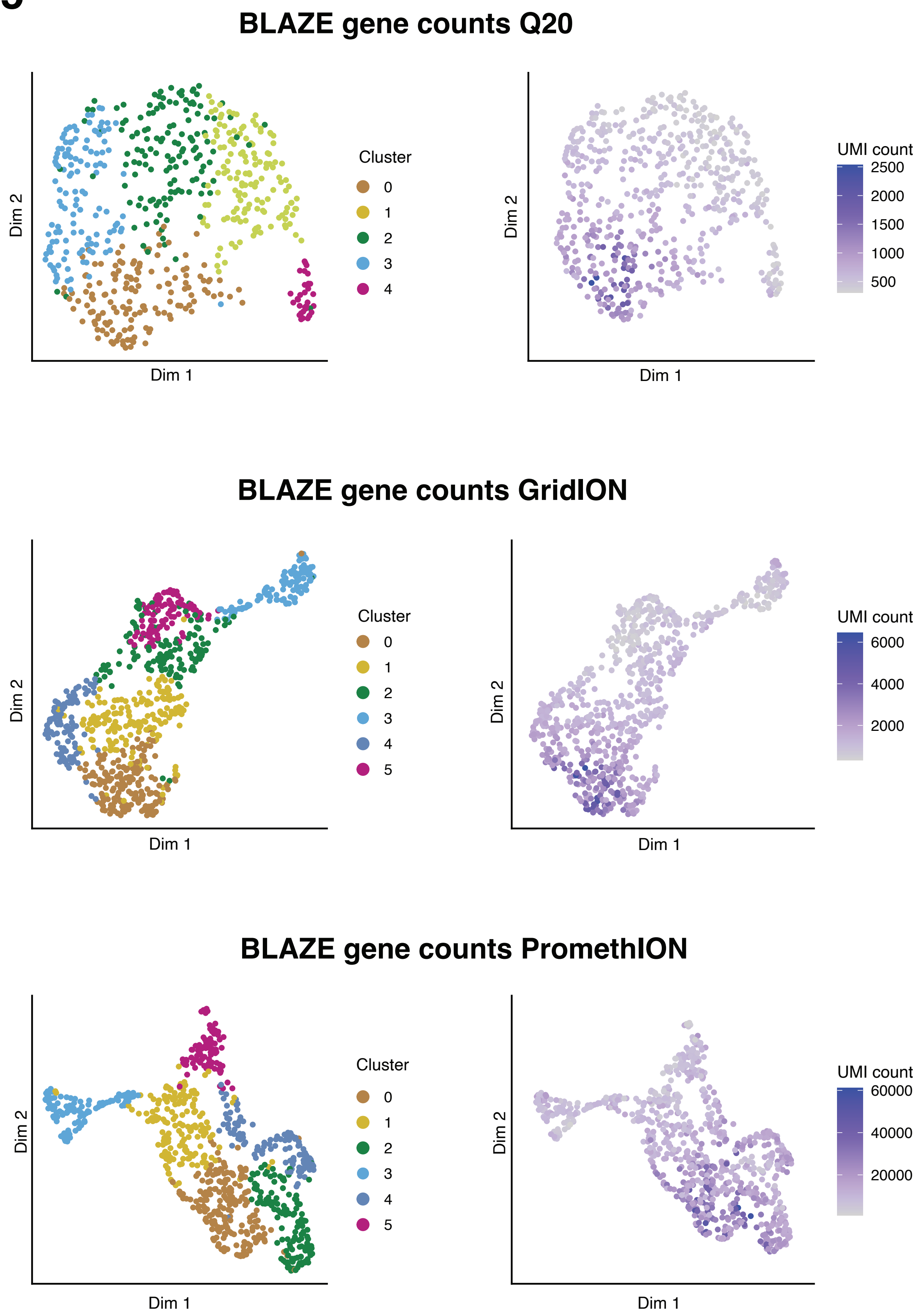

Fig. S5. Gene expression UMAP plot (using BLAZE whitelist) from PromethION, GridION and Q20 data. Gene expression is generated by FLAMES using BLAZE whitelist as input. Cells are coloured based on cluster (left) and UMI count (right).

Fig. S6

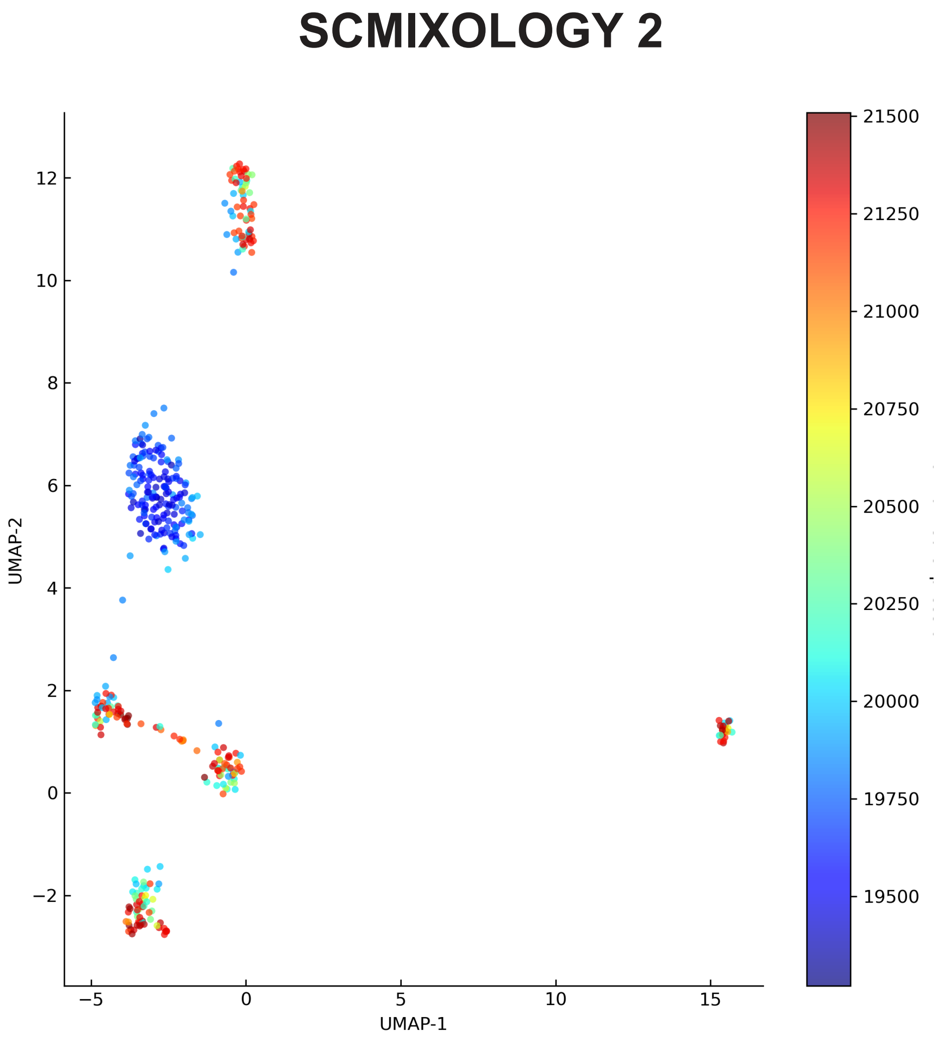

Fig. S6. Gene expression UMAP plot from Sockeye pipeline (scmixology 2 data). Cells are coloured based on normalised gene expression calculated within the Sockeye pipeline.

Fig. S7

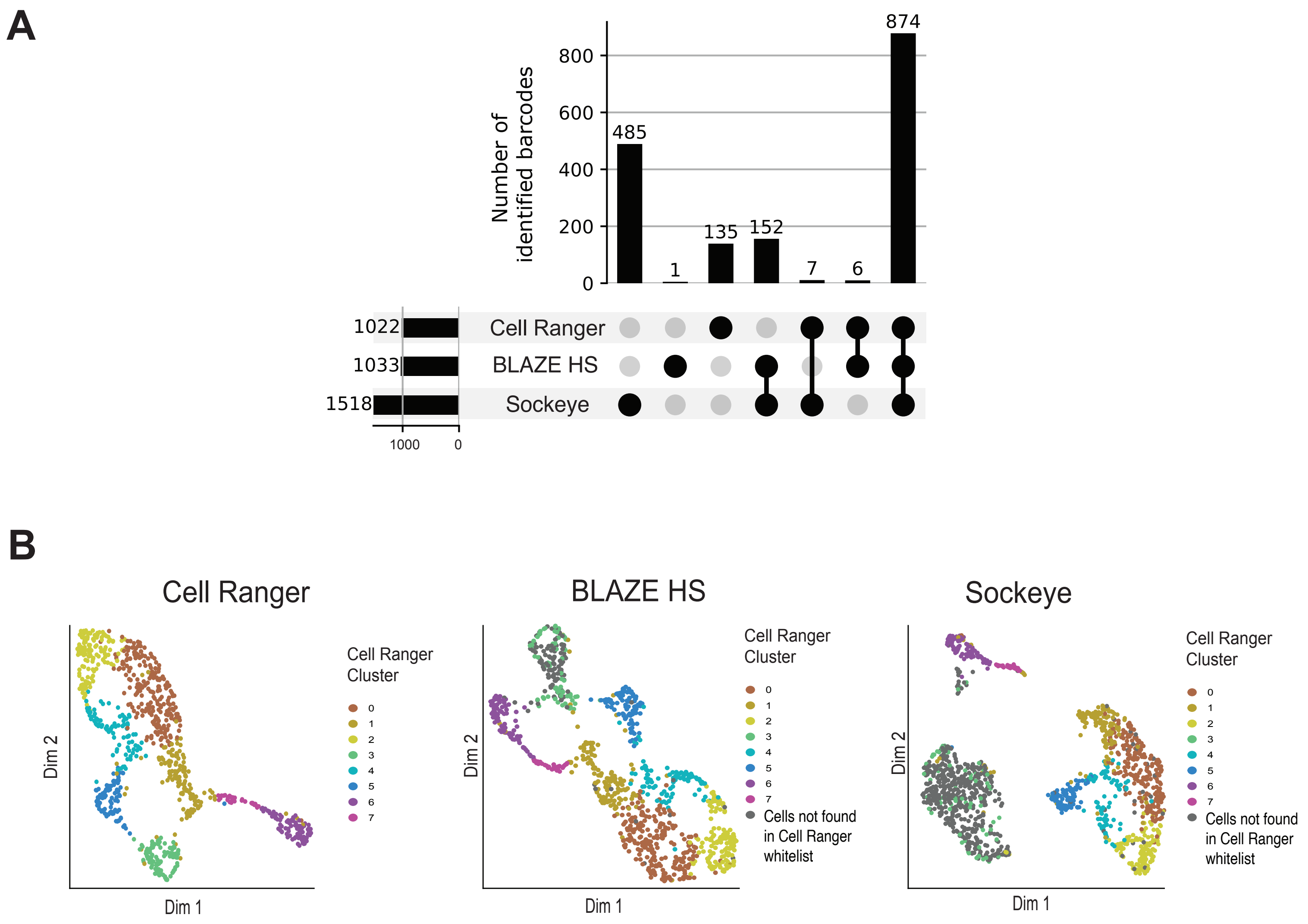

Fig. S7. Upset and UMAP plots for BLAZE HS mode without removing empty droplets. Using the PromethION data counts were generated with FLAMES with barcode whitelists from either Cell Ranger, Sockeye or BLAZE HS. **A:** Barcode upset plot comparing whitelists. Bar chart on left shows total number of barcodes found by each tool. Bar chart on top shows number of barcodes in the intersection of whitelists from specific combinations of methods. **B:** Isoform expression UMAP coloured by Cell Ranger clusters: Cells not found in Cell Ranger whitelist are coloured in gray.

Fig. S8

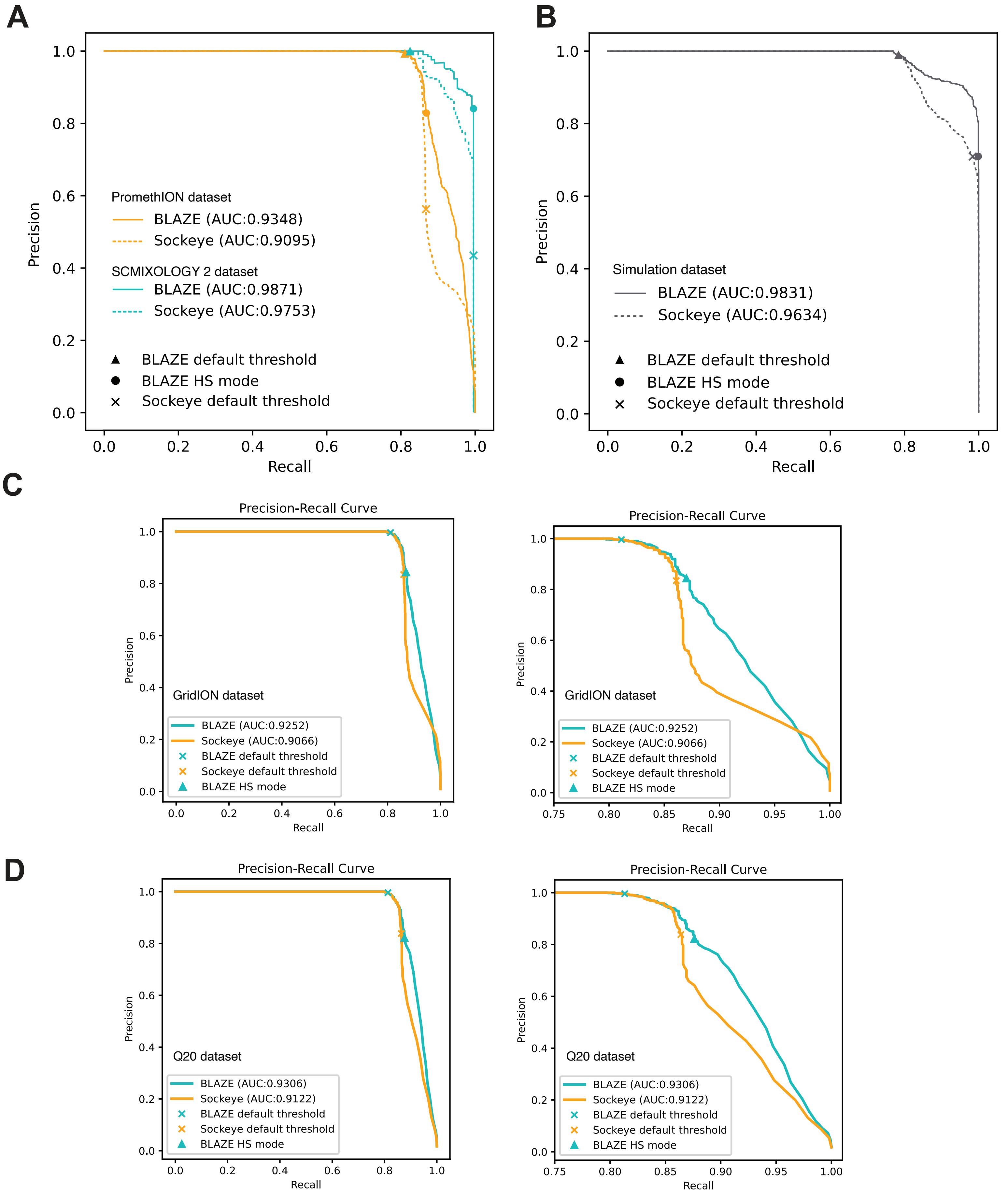

Figure S8: Full range of precision-recall curves across real and simulated data. A, C, D: real and B: simulated datasets, for BLAZE and Sockeye. A, C, D: Precision and recall were calculated across different count thresholds by using the barcodes identified from short reads (i.e. whitelist from Cell Ranger after removal of empty droplets) as the ground truth. **B:** As per A,C & D, except barcode whitelist represented a perfect ground truth. The numbers in the legend show area under the curve (AUC) values.

Fig. S9

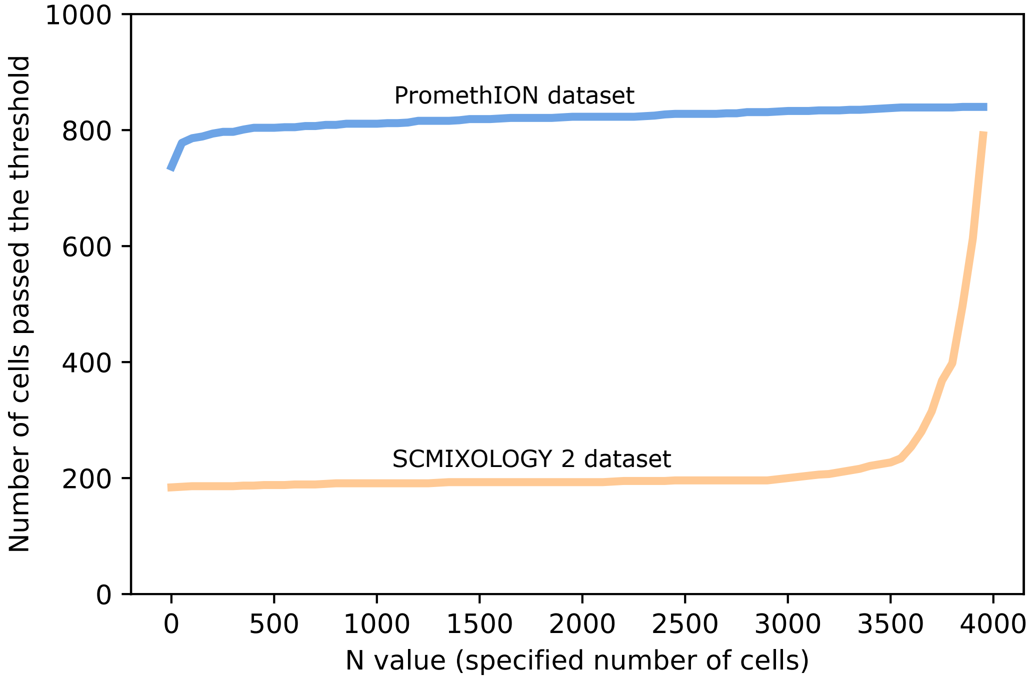

Fig. S9. Effect of specifying different numbers of expected cells in BLAZE. The count threshold in BLAZE is determined partly based on a specified number of cells (N). Figure shows how the number of cells passing this threshold (i.e. the identified number of cells) changes with different values of N in the PromethION and scmixology 2 datasets.
