# Supplementary material for: Identification of cell barcodes from long-read single-cell RNA-seq with BLAZE
Source: Genome Biol. 2023 Apr 6;24:66. doi: 10.1186/s13059-023-02907-y (PMC10077662; doi:10.1186/s13059-023-02907-y)
Supplement: Supplementary file 6 — Additional file 6. Review history. [file 13059_2023_2907_MOESM6_ESM.docx]

Review History

**First round of review**

**Reviewer 1**

**Were you able to assess all statistics in the manuscript, including the appropriateness of statistical tests used?** Yes, and I have assessed the statistics in my report.

**Were you able to directly test the methods?** Yes

**Comments to author:**

This manuscript presents the method BLAZE, which improves the accuracy of the barcode assignment for long-read Nanopore sequencing and then compares this method to Sockeye and Cell Ranger. While the manuscript is well written it does lack some key experiments to support their findings and glosses over some important considerations when discussing the results. The main issue that scientists face when they implement long-read single-cell sequencing is not necessarily barcode assignment because whitelisting can generate a set of putative barcodes using whitelisting. In fact this is exactly how the authors have addressed this issue and the authors should be commended on their code which seemed to be well written, although lacking some documentation in various parts. However, the major challenge for long-read single-cell sequencing is accurate UMI calling and the current manuscript does nothing to address this issue. I lay out my criticisms and comments below:

- Figure 1 is very nicely presented and works really well to explain the method.

- The method benchmarks itself against Cell ranger and Sockeye - however the correct benchmarking approaches are missing. The first droplet based methods in 2015 used species mixing experiments in order to, in part, measure the accuracy of their barcode assignment strategy. This has become the gold standard approach in the field in which to measure barcode assignment accuracy and is missing from this publication. The authors need to sequence a mixed species sample and then report the statistics on read miss assignment, as this can be determined by measuring the proportion of barcode mixing between the different cells from across both species.

- Substantially fewer barcodes are detected using BLAZE compared to cell ranger and Sockeye and they authors state that these are likely due to false barcode calling by Sockeye or CellRanger. The authors need to use the mixed species experiments to confirm this observation in a different system, as these cell barcodes are likely to be composed of mixed species.

- In the discussion the authors state that scCOLOR-seq and R2C2 methods have been used to improve barcode accuracy assignment, but that 10X chromium is more widely available. The advantages of both scCOLOR-seq and R2C2 approaches is not just to solve the barcode accuracy per se, but they allow UMIs to be more accurately assigned. This should be discussed and mentioned that BLAZE does not implement a method to improve UMI accuracy.

- It should be made clear to readers and potential users of this approach that this method should not be used to perform differential expression between cells or between different sets of samples because it does not improve UMI assignment which is still an issue with using 10X chromium.

- Aligned with the previous comment, the authors should evaluate the accuracy of their UMI assignment and overall sequencing accuracy per read. This could be done by extracting the primer sequence from the fastq file, determining the basecalling accuracy of this known region to the expected sequence and reporting a per read accuracy. The primer sequence could also be used to assess the accuracy of the UMI counts by assuming that all genes in each cell have a count == 1. Then running UMI correction tools (UMI-tools for example), to see if you observe a count greater than one. A count greater than one indicates a sequencing error and poor UMI accuracy correction.

- The authors mention split-seq as a potential application for long-read sequencing. However, because the method requires the user to fix the sample, it creates cross-links that are difficult (and in some cases impossible) to break, so this method is not realistically possible apply long-read sequencing to.

**Reviewer 2**

**Were you able to assess all statistics in the manuscript, including the appropriateness of statistical tests used?** There are no statistics in the manuscript.

**Were you able to directly test the methods?** Yes

**Comments to author:**

Summary

The paper presents a new computational tool, BLAZE, whose goal is to identify a whitelist of cellular barcodes from sequencing data. BLAZE targets recent single-cell library preparation techniques whereby RNA molecules are tagged with cellular barcodes (e.g. using 10x Chromium kit) and then these barcode-tagged RNA molecules are split into two pools: one for short-read sequencing and one for long-read sequencing. Previous methods (e.g. FLAMES and scTagger) identify the cellular barcodes present on the long-reads by using a whitelist of barcodes extracted from the short-reads. BLAZE's goal is to generate this whitelist from the long-reads directly and thus enabling a long-read only single-cell data generation and analysis. It's noteworthy that another tool, Sockeye, performs the same task as part of its pipeline.

Comments

- Unlike 10x Genomics Cell Ranger method of identifying barcodes of short-reads (which includes an error-correction step and a statistical test based on EmptyDrops for distinguishing valid barcode from ambient RNA barcodes), BLAZE's technical contribution is simple and potentially limited: poly-A tails and Oxford Nanopore adapters are identified on the long-reads, 16bps following the adapters are extracted as putative cellular barcodes if their quality scores are above a threshold, and the most frequent barcodes are kept as the whitelist. BLAZE thus seems to rely heavily on having the barcode of each cell correctly sequenced in its entirety at a very high frequency. This seems to be the view of the authors too: "With sufficient per-cell sequencing depth, this means each cell should be supported by large number of high-quality putative barcodes". However, it is unclear if this method, which involves no error-correction, can successfully identify cellular barcodes if the number of reads per cell is lower than what is used in the paper's experiments. And the presented datasets all have a very high coverage of reads per cell. Many practical datasets have an order of magnitude fewer long-reads per cell; would BLAZE perform as well on such datasets?

- The paper claims that BLAZE results in lower false positive barcode identification. However, this is not convincing. BLAZE's approach seems to be more conservative when including barcodes into its whitelist than Cell Ranger or Sockeye. It makes sense in this stage to err on the side of including barcodes than on excluding them. This is because these barcodes still need to be matched against each of the long-reads, and any barcode with very low long-read count should be filtered as a clustering preprocessing step (as is typical of barcodes with too low short-read count or gene count). It also makes sense to use Cell Ranger's whitelist of barcodes as ground truth in the absence of simulated data. When doing so, BLAZE seems to miss a lot of Cell Ranger whitelisted barcodes (Fig 1C and S4) and Sockeye seems to capture much more of Cell Ranger's whitelist. This closer concordance between Sockeye and Cell Ranger seems to result in better clustering similarity as well, with a whole cluster in common between them that is missing from BLAZE: Fig 2A/B shows that cluster 3 is present in Cell Ranger and Sockeye but missing from BLAZE. The authors' main evidence for this cluster being representative of false positive barcode detections is having "low UMI and gene/isoform counts". If the expression is indeed too low, then this is easily remedied by preprocessing barcodes for the extremely low UMI counts as is typical in dataset preparation for clustering and thus should not be part of comparing Cell Ranger/Sockeye and BLAZE. Otherwise, I do not believe having low UMI/gene count is evidence for these cell barcodes to be a result of false positive detections.

- The paper mentions that the authors "identified significant differences in the expression of hundreds of genes including key transcription factors such as ELAVL4 and NHLH1 (Fig. 3)" in one of BLAZE's clusters. Since all of BLAZE's clusters are captured also by Cell Ranger and Sockeye, this experiment does not provide evidence of BLAZE being better than Cell Ranger or Sockeye. Perhaps it would be more informative to perform similar analysis for the cluster that is differentially missing from BLAZE and present in the other methods.

- I tested BLAZE on an inhouse single-cell transcriptomic LR dataset that has ~7M pass reads. BLAZE terminated in 8.5 hours, processing on average 225 reads per second. BLAZE used a maximum RAM of 43.40GB. This very large memory and time demand for a simple preprocessing step is prohibitive (especially memory) for many labs especially if multiple files are to run concurrently.

**Reviewer 3**

**Were you able to assess all statistics in the manuscript, including the appropriateness of statistical tests used?** Yes, and I have assessed the statistics in my report.

**Were you able to directly test the methods?** Yes

**Comments to author:**

In this work, the authors present the long-read single-cell RNA-seq tool BLAZE. BLAZE aims to improve upon single-cell barcode detection using only long-reads alone instead of combining short-read and long-read data. BLAZE works by identifying putative barcodes in single-cell reads and whitelisting those which pass a quantile-based count threshold. The authors show that BLAZE achieves comparable accuracy to existing methods that use short-read RNA-Seq data. Unlike other tools (Cell Ranger and Sockeye), BLAZE does not white list barcodes that are poorly supported by long-reads and the authors show that BLAZE is robust to changes in sequencing depth when using default parameters. The authors demonstrate that BLAZE is more conservative compared to Sockeye, and they show that this conservative approach to barcode detection is better for downstream analysis. While the authors show that the overall performance appears to be better than Sockeye, the difference appears to only be relevant for barcodes that are very noisy and which are below the threshold. Therefore, the results suggest that a similar conservative threshold applied to Sockeye would result in almost identical results to BLAZE.

Unlike Sockeye, BLAZE is a dedicated tool for barcode detection and whitelisting. Furthermore, Sockeye is not described in a scientific publication, therefore BLAZE would be the first peer-reviewed and fully evaluated barcode detection tool that uses long-read data alone. Together with the good performance, BLAZE is a very useful and timely contribution to the field, which will help to simplify the sequencing and analysis of long-read single-cell workflows going forward. BLAZE could be successfully downloaded and run from GitHub, only encountering a few easily circumventable minor typos in their online documentation.

Please find my comments below, which I believe once addressed will improve the robustness of the evaluation of BLAZE compared to Sockeye and Cell Ranger.

Major comments:

1) Some of the claims in the manuscript are too strong and do not accurately represent the results. In particular (1) many claims are specific to the thresholds that are used, with BLAZE using a more conservative threshold than Sockeye. The authors show that the conservative threshold appears to be more useful, but this does not indicate that the method in BLAZE is more accurate. (2) Evaluation of barcode detection methods should be primarily based on the ability to detect barcodes, not on the quality of the cells associated with the barcodes at a specific threshold, which some users might prefer to address through filtering of cells based on expression data. Specific examples and comments:

* In this work it is claimed "BLAZE outperforms existing tools". The majority of the results compare BLAZE and Sockeye at one set of parameters. At these parameters BLAZE is shown to be more conservative which leads to clearer outcomes in the clustering. However this does not prove BLAZE is outperforming Sockeye on the task of barcode detection, as Sockeye may be able to achieve these same results with more stringent thresholds. When the thresholds are compared in Fig.6 a minor increase in the AUC is seen for BLAZE. It is possible that BLAZE only outperforms Sockeye for thresholds that are lower than BLAZE's default parameters. Therefore, there are no results to show that in practice BLAZE outperforms Sockeye when comparable thresholds are used.

* To address this, the authors should update Figure 6 to highlight the point on each line that corresponds to the default thresholds for BLAZE and Sockeye. Furthermore Figure 6 should be shown as the full range of recall, with the zoomed in version as an addition. The current visualisation is helpful, but overstates the difference between methods.

* Depending on these results, the authors should adjust their claims.

* Figure 2/Table 2 could be reproduced using more conservative and comparable parameters for Sockeye. This indicates that a conservative threshold is useful, which is valid for both Sockeye and BLAZE

* The authors claim that "[BLAZE] provides a more accurate representation of the cells present in LR scRNA-seq than using matched short-reads". There are no results that evaluate the performance of BLAZE against Cell Ranger. The cells/barcodes that are detected by Cell Ranger may represent true cells (albeit low quality ones) and therefore the comparison of BLAZE with cell ranger does not indicate higher accuracy for the task of identifying barcodes, but that BLAZE has a more stringent criteria for barcode filtering which removes barcodes that represent cells of low quality. Either additional results need to be provided that compare Blaze against matched short-reads that show it is indeed more accurate or this claim needs to be adjusted.

2) Usability of BLAZE

The usability of BLAZE is an important factor. Currently the authors represent the runtimes using 32 cores. Could the authors provide more information on running BLAZE with less cores and the memory requirements. A quantified comparison of the memory usage and run time to do the full workflow with Cell Ranger and Sockeye at the same specifications is also important to support BLAZE despite it not being a fair comparison due to the additional tasks in Sockeye. The authors could compare the performance of BLAZE to using this python script if they are concerned about an unfair comparison (https://github.com/epi2me-labs/wf-single-cell/blob/master/subworkflows/process_bams.nf)

Minor Comments:

* Sockeye is deprecated and replaced: https://github.com/epi2me-labs/wf-single-cell

* BLAZE is linked to FLAMES. Would it be an option to create an output that other tools such as StringTie2 (used in Sockeye) could use (eg. de-multiplexed fastq files). That could increase adaptation of BLAZE since most downstream tools for long read single cell methods are not yet established.

* In the evaluation the authors use data from Tian et al (2021) where barcodes were assigned to specific cell lines. It would be beneficial to readers for the authors to briefly summarise how barcodes were assigned to each cell line, as the method could have intersected with one of the applied tools creating a bias (it doesn't, but unless a reader looks up another paper the uncertainty is there).

* "These results suggested that Cell Ranger and Sockeye may be consistently identifying false positive long-read barcodes" - As Cell Ranger does not detect false positive long-read barcodes, this is incorrect to say.

There are several observations made when comparing BLAZE with Cell Ranger and Sockeye and it would be good if the authors could speculate as to their origin in either the results of discussion:

* The authors suggest the higher numbers of white-listed barcodes in Cell Ranger may be because these cells exist in SR data but are poorly represented amongst long-reads. Can the authors speculate why this would happen? If the reason is due to sequencing depth, why would there not be a change in the number of accepted barcodes between the PromethION and GridION runs? This could be partially explained with Barcode rank plots (knee plots) of the GrioION and Q20 runs.

* BLAZE and Sockeye find 5 barcodes that are not in the short read data. Could the authors speculate on how this could occur?

**Authors Response**

**Point-by-point responses to the reviewers’ comments:**

Our thanks to the reviewers for their useful and constructive comments on our manuscript. As requested, we have performed a number of additional analyses to improve the paper. This included creating a new software pipeline (SLSim) to simulate nanopore single-cell reads, and new analyses of simulated data, “empty droplets”, and barcode edit distances. These all support our central finding that BLAZE has superior performance to Sockeye and identifies fewer false positive barcodes. In addition, we have added a “high sensitivity” mode and “background barcode output” function to BLAZE to allow users to increase recall and perform more sophisticated analyses of which barcodes represent cells vs ambient/empty droplets. The computational performance of BLAZE has also been improved with faster runtimes and much lower memory requirements. We believe we have successfully addressed all reviewer concerns and hope the manuscript will now be suitable for publication in Genome Biology.

Reviewer comments are shown in black. Our responses are in dark blue.

**Reviewer reports:**

**Reviewer #1:** This manuscript presents the method BLAZE, which improves the accuracy of the barcode assignment for long-read Nanopore sequencing and then compares this method to Sockeye and Cell Ranger. While the manuscript is well written it does lack some key experiments to support their findings and glosses over some important considerations when discussing the results. The main issue that scientists face when they implement long-read single-cell sequencing is not necessarily barcode assignment because whitelisting can generate a set of putative barcodes using whitelisting. In fact this is exactly how the authors have addressed this issue and the authors should be commended on their code which seemed to be well written, although lacking some documentation in various parts. However, the major challenge for long-read single-cell sequencing is accurate UMI calling and the current manuscript does nothing to address this issue. I lay out my criticisms and comments below:

As long-read single cell sequencing is a new and evolving technique there are a number of challenges that need addressing. One of these is accurate determination of the cell barcodes. Requiring matched short-read sequencing to supply these adds time and cost and, as we find, short-read barcodes are not a perfect ground truth set for long-read libraries. Therefore, we aimed to solve this issue with BLAZE. UMI calling is another challenge, but not the one we set out to solve. Thus, creating a new tool to improve UMI calling is outside the aims and scope of this work. In addition, there are a number of programs that address UMI calling, and we find FLAMES (Tian *et al*, 2021) performs quite well at this task. BLAZE does not seek to partially replicate the function of FLAMES but instead provide accurate barcode whitelists that can act as standard inputs into this and other programs.

- Figure 1 is very nicely presented and works really well to explain the method.

Thank you, we are pleased it is informative.

- The method benchmarks itself against Cell ranger and Sockeye - however the correct benchmarking approaches are missing. The first droplet based methods in 2015 used species mixing experiments in order to, in part, measure the accuracy of their barcode assignment strategy. This has become the gold standard approach in the field in which to measure barcode assignment accuracy and is missing from this publication. The authors need to sequence a mixed species sample and then report the statistics on read miss assignment, as this can be determined by measuring the proportion of barcode mixing between the different cells from across both species.

&

- Substantially fewer barcodes are detected using BLAZE compared to cell ranger and Sockeye and the authors state that these are likely due to false barcode calling by Sockeye or CellRanger. The authors need to use the mixed species experiments to confirm this observation in a different system, as these cell barcodes are likely to be composed of mixed species.

We thank the reviewer for their suggestions on how to benchmark BLAZE against a proper ground truth dataset. Based on the suggestions from all 3 reviewers we have now also benchmarked BLAZE using simulated data as species mixing experiments are not able to ascertain the precision and recall of BLAZE for barcode determination (explained in detail below). The results from the simulated data confirm the results we see in our real data analysis. Sockeye has a lower precision (ie: it identifies more false positive barcodes) than BLAZE across experimentally desirable recall thresholds. We have updated the manuscript (Methods, Results section “Overall comparison between BLAZE and Sockeye”, Figures 7 and S8 and Discussion) to incorporate these additional results.

The relevant results section now reads:

“Using real data it is not possible to generate a perfect ground truth barcode whitelist, even after removing identifiable empty droplets from the Cell Ranger whitelist. Therefore, we created a software pipeline SLSim (https://github.com/youyupei/SLSim) to simulate nanopore single cell long-reads, allowing us to compare BLAZE and Sockeye with a known ground truth barcode whitelist. Precision-recall curves across different count thresholds confirmed BLAZE consistently outperforms Sockeye (**Fig. 7B, Additional File 1:** **Fig**. **S8B**).”

Limitations with species mixing experiments:

The 2015 droplet papers (Klein *et al*., 2015, Macosko *et al.,* 2015), referenced by the reviewer used species mixing to check their methods had only 1 cell in each droplet (as cases where 2 cells were present in a droplet would commonly give rise to a single barcode that had reads mapping to multiple species). This experiment was not performed to check the accuracy of barcode detection itself and cannot do so, as by far the most likely explanation for a barcode having reads coming from multiple species is not barcode mis-assignment, but multiple cells in the droplet. Many other single cell papers, (including the original 10x paper Zheng et al, 2017) have used species mixing this way and to the best of our knowledge is it not used for the purpose requested by the reviewer.

In addition, even if one could eliminate having multiple cells in a droplet, for example by utilising existing single cell sequencing datasets from different species and mixing them computational, there is a further problem. The purpose of BLAZE is to identify an accurate list of barcodes present in a sample (the so-called whitelist). BLAZE does not assign sequencing reads to barcodes. This is done by downstream tools. Therefore even if BLAZE perfectly identifies the barcodes present, a downstream tool could mis-assign a read from one species to a barcode from the other. Such a case would not provide any information on the performance of BLAZE. What matters for benchmarking BLAZE is the accuracy and completeness of its barcode whitelist. This is best determined when the ground truth is absolutely known, which requires simulated data.

- In the discussion the authors state that scCOLOR-seq and R2C2 methods have been used to improve barcode accuracy assignment, but that 10X chromium is more widely available. The advantages of both scCOLOR-seq and R2C2 approaches is not just to solve the barcode accuracy per se, but they allow UMIs to be more accurately assigned. This should be discussed and mentioned that BLAZE does not implement a method to improve UMI accuracy.

We have edited the manuscript to reiterate which steps are performed by BLAZE and that UMI calling (and additional downstream analysis steps) are performed by other programs (in this case FLAMES).

The relevant text now reads:

“We designed BLAZE to seamlessly integrate with the existing FLT-seq - FLAMES pipeline which performs UMI calling, read assignment and mapping to enable identification and quantification of RNA isoforms and their expression profiles across individual cells and cell-types.”

- It should be made clear to readers and potential users of this approach that this method should not be used to perform differential expression between cells or between different sets of samples because it does not improve UMI assignment which is still an issue with using 10X chromium.

As BLAZE does not perform differential expression analyses, whether or not researchers performing LR scRNA-seq choose to perform differential expression analyses will depend on their confidence in the other tools which carry out these functions. To ensure readers understand the remaining challenges in LR scRNA-seq analysis we have updated our discussion to address these issues more specifically. An additional discussion paragraph now reads:

“While LR scRNA-seq is becoming more feasible as sequencing outputs increase and analysis packages are designed, not all analysis challenges are completely solved. Along with barcode identification; the identification and collapse of UMIs; assignment of reads to identified barcodes; and construction of accurate isoforms are all areas of active research. Further progress in these areas will be important to enable higher accuracy isoform identification, quantification and differential expression analyses from LR scRNA-seq data.”

- Aligned with the previous comment, the authors should evaluate the accuracy of their UMI assignment and overall sequencing accuracy per read. This could be done by extracting the primer sequence from the fastq file, determining the basecalling accuracy of this known region to the expected sequence and reporting a per read accuracy. The primer sequence could also be used to assess the accuracy of the UMI counts by assuming that all genes in each cell have a count == 1. Then running UMI correction tools (UMI-tools for example), to see if you observe a count greater than one. A count greater than one indicates a sequencing error and poor UMI accuracy correction.

UMI calling in this study was performed with FLAMES, which was previously published by Tian et al (2021) in Genome Biology. FLAMES uses a sophisticated method to separate each isoform from each gene and then clusters and collapses UMIs while allowing for sequencing errors (using edit distance-based clustering).

As BLAZE does not perform UMI assignment, the absolute accuracy of UMI assignment in downstream tools is not critical to the results of this manuscript or to the evaluation of BLAZE. In addition, while the evaluation method suggested by the reviewer is innovative, we don’t believe its results would be comparable to UMI assignment accuracy. The error profile and number of errors within primer sequences would likely be different to UMIs due to differences in sequence length (primer sequence is longer); nucleotide composition (primer sequences are identical, UMIs are not and nanopore errors are sequence context dependent) and the fact there is often an increased read error rate near at the start of nanopore reads which would affect the primer region more than the UMI region.

Note: The average sequencing accuracy for each dataset is reported in **Table 1**.

- The authors mention split-seq as a potential application for long-read sequencing. However, because the method requires the user to fix the sample, it creates cross-links that are difficult (and in some cases impossible) to break, so this method is not realistically possible apply long-read sequencing to.

Long-read split-seq was published in 2021 in Genome Biology (Rebboah et al), it is cited in our manuscript as an example of a long-read scRNA-seq method which currently uses matched short-reads to identify barcodes. Our existing text reads:

“while LR nanopore protocols for other scRNA-seq modalities such as Split-seq are also now available”

**Reviewer #2: Summary**

The paper presents a new computational tool, BLAZE, whose goal is to identify a whitelist of cellular barcodes from sequencing data. BLAZE targets recent single-cell library preparation techniques whereby RNA molecules are tagged with cellular barcodes (e.g. using 10x Chromium kit) and then these barcode-tagged RNA molecules are split into two pools: one for short-read sequencing and one for long-read sequencing. Previous methods (e.g. FLAMES and scTagger) identify the cellular barcodes present on the long-reads by using a whitelist of barcodes extracted from the short-reads. BLAZE's goal is to generate this whitelist from the long-reads directly and thus enabling a long-read only single-cell data generation and analysis. It's noteworthy that another tool, Sockeye, performs the same task as part of its pipeline.

Comments

- Unlike 10x Genomics Cell Ranger method of identifying barcodes of short-reads (which includes an error-correction step and a statistical test based on EmptyDrops for distinguishing valid barcode from ambient RNA barcodes), BLAZE's technical contribution is simple and potentially limited: poly-A tails and Oxford Nanopore adapters are identified on the long-reads, 16bps following the adapters are extracted as putative cellular barcodes if their quality scores are above a threshold, and the most frequent barcodes are kept as the whitelist. BLAZE thus seems to rely heavily on having the barcode of each cell correctly sequenced in its entirety at a very high frequency. This seems to be the view of the authors too: "With sufficient per-cell sequencing depth, this means each cell should be supported by a large number of high-quality putative barcodes". However, it is unclear if this method, which involves no error-correction, can successfully identify cellular barcodes if the number of reads per cell is lower than what is used in the paper's experiments. And the presented datasets all have a very high coverage of reads per cell. Many practical datasets have an order of magnitude fewer long-reads per cell; would BLAZE perform as well on such datasets?

BLAZE provides comparable performance on high and low read-depth datasets and its results are largely insensitive to read-depth. In addition, we have added an option to BLAZE which allows EmptyDrops correction of BLAZE barcodes (covered in more detail below and in manuscript section: “Increasing barcode recall with BLAZE”.

The performance of BLAZE on datasets of different reads depths is a crucial point. We address this in the Results section “Barcode detection with BLAZE is robust to changes in read depth or read accuracy”, as well as Table 1 and Table 2. These show the sequence depth and BLAZE barcode detection results for each dataset. Our datasets range from ~3,300 pass reads per cell (Q20) to >60k (PromethION). BLAZE produces almost identical results (between 802 & 804 barcodes) for each. The Q20 dataset would not be considered “very high coverage” for single cell experiments, especially for those that aim to investigate isoforms. Even in the context of short reads ~3,300 reads per cell would be considered well below the minimum of 20,000 paired end reads per cell recommended by [10x Genomics](https://kb.10xgenomics.com/hc/en-us/articles/115002022743-What-is-the-recommended-sequencing-depth-for-Single-Cell-3-and-5-Gene-Expression-libraries-) for studying genes. While we don’t believe a further order-of-magnitude reduction in read depth would generate very informative data on gene and isoform expression or cell clusters, we have tested BLAZE performance by downsampling the Q20 data to a mean of ~500 reads per cell. Here, BLAZE identified 812 barcodes which was almost identical to the above results.

We have added this information to the main text:

“We also downsampled the Q20 data to an average of 500 reads per cell to test the performance of BLAZE when there are very low numbers of reads per cell. BLAZE returned an almost identical 812 barcodes, demonstrating it performs consistently across data sets with variable read depths and different sequencing accuracies.”

In addition, we have updated Table 1 to include the “Mean reads per cell” in each sequencing dataset to make this clearer to readers.

- The paper claims that BLAZE results in lower false positive barcode identification. However, this is not convincing. BLAZE's approach seems to be more conservative when including barcodes into its whitelist than Cell Ranger or Sockeye. It makes sense in this stage to err on the side of including barcodes than on excluding them. This is because these barcodes still need to be matched against each of the long-reads, and any barcode with very low long-read count should be filtered as a clustering preprocessing step (as is typical of barcodes with too low short-read count or gene count). It also makes sense to use Cell Ranger's whitelist of barcodes as ground truth in the absence of simulated data. When doing so, BLAZE seems to miss a lot of Cell Ranger whitelisted barcodes (Fig 1C and S4) and Sockeye seems to capture much more of Cell Ranger's whitelist. This closer concordance between Sockeye and Cell Ranger seems to result in better clustering similarity as well, with a whole cluster in common between them that is missing from BLAZE: Fig 2A/B shows that cluster 3 is present in Cell Ranger and Sockeye but missing from BLAZE. The authors' main evidence for this cluster being representative of false positive barcode detections is having "low UMI and gene/isoform counts". If the expression is indeed too low, then this is easily remedied by preprocessing barcodes for the extremely low UMI counts as is typical in dataset preparation for clustering and thus should not be part of comparing Cell Ranger/Sockeye and BLAZE. Otherwise, I do not believe having low UMI/gene count is evidence for these cell barcodes to be a result of false positive detections.

We have performed a number of additional analyses and added new features to BLAZE to address these various interlinked points. These include: addition of simulated data, which supports the superior performance of BLAZE over Sockeye; empty droplets and edit distance analyses which confirm many Sockeye (and some Cell Ranger) barcodes do not denote true cells; a BLAZE High Sensitivity Mode to provide more permissive barcode identification should the user require it; and an analysis to show how the prevalence of read mis-assignment to erroneous barcodes means it is better to filter out these barcodes at the whitelist generation step. We discuss these in detail below and in the revised manuscript.

**Comparison of BLAZE and Sockeye using simulated Data**

As there was no software that could simulate nanopore single cell data we have created a new software pipeline called SLSim (<https://github.com/youyupei/SLSim>). Using simulated data with a set of known ground truth cell barcodes we find BLAZE outperforms Sockeye in a precision-recall analysis. The precision of BLAZE is superior to Sockeye over a range of recall values showing Sockeye identifies a higher proportion of false positive barcodes. See Section “Overall comparison between BLAZE and Sockeye” and Figures 7 and S8.

**Analyses of real data demonstrates false positive barcode detections by Sockeye**

In the manuscript we find that Sockeye identifies false positive barcodes and provided low UMI and feature counts as evidence for this claim. We find this both for our own data and also in the scmixology2 data (Figure 5). The latter data contains cells from 5 cells lines and hence should only produce 5 clusters. This is seen in the BLAZE results and the original scmixology2 short-read scRNA-seq. However long-read scRNA-seq with barcodes from Cell Ranger and Sockeye shows >5 cell clusters, which is strong evidence both methods are providing barcodes which do not correspond to sequenced cells in the long-read data. Thus, higher concordance between Sockeye and Cell Ranger and/or identification of additional clusters is not necessarily evidence of better performance and can be evidence of the opposite.

To further support our claims we have performed two additional analyses, which we describe below. Both of these demonstrate Sockeye identifies empty droplets and false positive cells derived from sequencing errors. Both analyses have been added to the manuscript. See updated Figures 2 and 5 and sections:

**“Additional cell cluster found with Sockeye and Cell Ranger barcodes corresponds to empty droplets and barcodes with sequencing errors”**

&

“**BLAZE correctly identifies barcodes in long read single-cell data of known cell lines”**

Analysis of Empty drops:

Using the R package DropletUtils we identified ‘cells’ that are empty droplets and have an ambient RNA profile. 211 (15%) of the ‘cells’ identified by Sockeye in the PromethION data set and 47 (11%) in the scmixology2 data are empty droplets. These almost exclusively belong to the cluster not found by BLAZE. These barcodes have been included in the sockeye whitelist due to their high counts but would be considered uninformative. In contrast BLAZE output close to 0 empty droplets

Edit distance of Sockeye only barcodes to true Cell Ranger barcodes:

We calculated the percentage of barcodes from the Sockeye only cells that have an edit distance (ED) ≤ 2 when compared to the Cell Ranger whitelist. We find that ~60% of Sockeye-only cells have an ED ≤ 2 which is about 60x higher than a random set of 10x barcodes. The much higher than expected proportion of Sockeye only barcodes that are very similar to the barcodes of real cells is almost certainly explained by them being false positive detections derived due to nanopore sequencing errors. Coloring the UMAP plots with the ‘cells’ that are empty droplets or have an ED ≤ 2 demonstrates clearly that the unique sockeye cluster is primarily composed of empty droplets or barcodes with errors.

**Prevalent read mis-assignment supports filtering out erroneous barcodes at the whitelist stage:**

Reviewer 2 suggests that “It makes sense in this stage to err on the side of including barcodes than on excluding them”. However, in circumstances where noisy long reads routinely cause errors in the barcode sequence it becomes increasingly difficult to distinguish between true barcodes and barcodes generated from sequencing errors. Barcodes generated from sequencing errors are similar in sequence to real barcodes as shown above. This similarity means sequencing reads are assigned to them and some can have the expression profiles of true cells. This is seen in Figure 2, where some Sockeye only barcodes are present in the clusters found by all methods. The ‘cells’ have an edit distance indicative of false positives suggesting their expression profiles are due to read miss-assignment. Therefore, we believe it is best to remove such barcodes at the stage of generating the whitelist.

We further investigated the impact of read mis-assignment by testing the reviewer's suggestion to apply a post filtering threshold based on low read or feature counts. Filtering out all cells with fewer than 1,000 isoforms in the scmixology2 data set means discarding ~7% of all mapped reads (which also acts as an estimate for the number of reads assigned to barcodes that do not represent real cells). Sockeye now only identifies 8 cells that are not found in the matched short read data, however all 8 of these barcodes have an edit distance ≤ 2 meaning they are likely false positives due to nanopore sequencing errors. Our data therefore demonstrate that even using highly stringent filtering thresholds Sockeye has errors. In addition, filtering at this stage, instead of producing an accurate barcode whitelist, causes the loss of otherwise usable reads. Given these data we argue that using BLAZE produces better results by default, without the need for a highly stringent filtering threshold. We also note that it can be difficult to determine the filtering criteria as it will likely vary across cell type and sequencing dept. Full workflow of this analysis can be found here: <https://github.com/youyupei/bc_whitelist_analysis/blob/main/docs/empty_drops_scmixology_-1000ft_sockeye.html>.

We have also expanded discussion of these issues in the manuscript including the discussion paragraph beginning: “The accurate identification of single-cell barcodes is crucial to downstream gene and isoform quantification.”

**BLAZE High Sensitivity mode:**

In order to address the concerns of Reviewer 2 we have implemented a high sensitivity (HS) mode for BLAZE which can be used if a user prioritises recall over precision (see **Fig. 6, 7, S7 and new section: Increasing barcode recall with BLAZE**). Furthermore, BLAZE can now also output a list of background barcodes that can be used in the empty drops workflow to further improve recall performance, while still maintaining superior performance to Sockeye. We thank the Reviewer for this comment as users now have an option to run BLAZE in two modes, broadening the use case for the software which we feel is an improvement.

- The paper mentions that the authors "identified significant differences in the expression of hundreds of genes including key transcription factors such as ELAVL4 and NHLH1 (Fig. 3)" in one of BLAZE's clusters. Since all of BLAZE's clusters are captured also by Cell Ranger and Sockeye, this experiment does not provide evidence of BLAZE being better than Cell Ranger or Sockeye. Perhaps it would be more informative to perform similar analysis for the cluster that is differentially missing from BLAZE and present in the other methods.

We performed the differential expression analysis to verify that clustering cells with a high confidence set of barcodes from BLAZE derives logical and biologically relevant data. The point here was not a comparison between methods but a demonstration that the clusters produced from BLAZE whitelist are informative.

To address the reviewers’ query, we have performed the same analysis on the Cell Ranger and Sockeye clusters that are not found in BLAZE. The differential expression results can be found in **Additional file 4**. However, as covered in the updated analysis and in this response, this analysis is for the most part uninformative for the following reasons

1. Many “cells” in these unique clusters are empty droplets containing an ambient RNA profile. Such profiles will not provide meaningful differential expression information.
2. We show that a very large proportion (~60%) of Sockeye-only barcodes are very similar (edit distance =< 2) to Cell Ranger true barcodes and are likely false positives. Highly similar barcodes cause ambiguous read assignment and although these reads are derived from a real cell, ambiguous assignment can give a misleading impression of differential expression.

We have added the following text to the manuscript:

“In contrast, marker gene analysis on the additional clusters found using Cell Ranger and Sockeye barcodes found 7 and 8 DE genes respectively (**Additional file 4: Table S3, S4)**. No biological relevance could be assigned to these expression changes, in line with these clusters largely representing empty droplets and non-cell associated barcodes.”

- I tested BLAZE on an inhouse single-cell transcriptomic LR dataset that has ~7M pass reads. BLAZE terminated in 8.5 hours, processing on average 225 reads per second. BLAZE used a maximum RAM of 43.40GB. This very large memory and time demand for a simple preprocessing step is prohibitive (especially memory) for many labs especially if multiple files are to run concurrently.

We thank the reviewer for testing BLAZE and agree that usability and processing time for LR data sets is a high priority for BLAZE. We have addressed the concerns of the reviewer by updating the software and now BLAZE automatically performs multiprocessing even with a single fastq file, which makes it much faster and more efficient. The memory requirement is significantly reduced in the updated version. BLAZE has been tested again on a data set containing 10M pass reads using 6 CPUs. BLAZE terminated after 1.5 hours and used ~1.5 GB of RAM. We have also run re-run BLAZE using 6 CPUs on all the data sets in the manuscript and have updated **additional file 5** with runtime data and RAM usage.

**Reviewer #3:**

In this work, the authors present the long-read single-cell RNA-seq tool BLAZE. BLAZE aims to improve upon single-cell barcode detection using only long-reads alone instead of combining short-read and long-read data. BLAZE works by identifying putative barcodes in single-cell reads and whitelisting those which pass a quantile-based count threshold. The authors show that BLAZE achieves comparable accuracy to existing methods that use short-read RNA-Seq data. Unlike other tools (Cell Ranger and Sockeye), BLAZE does not white list barcodes that are poorly supported by long-reads and the authors show that BLAZE is robust to changes in sequencing depth when using default parameters. The authors demonstrate that BLAZE is more conservative compared to Sockeye, and they show that this conservative approach to barcode detection is better for downstream analysis. While the authors show that the overall performance appears to be better than Sockeye, the difference appears to only be relevant for barcodes that are very noisy and which are below the threshold. Therefore , the results suggest that a similar conservative threshold applied to Sockeye would result in almost identical results to BLAZE.

Unlike Sockeye, BLAZE is a dedicated tool for barcode detection and whitelisting. Furthermore, Sockeye is not described in a scientific publication, therefore BLAZE would be the first peer-reviewed and fully evaluated barcode detection tool that uses long-read data alone. Together with the good performance, BLAZE is a very useful and timely contribution to the field, which will help to simplify the sequencing and analysis of long-read single-cell workflows going forward. BLAZE could be successfully downloaded and run from GitHub, only encountering a few easily circumventable minor typos in their online documentation.

We thank the reviewer for their support. We believe we have now fixed these minor typos on Github.

Please find my comments below, which I believe once addressed will improve the robustness of the evaluation of BLAZE compared to Sockeye and Cell Ranger.

Major comments:

1) Some of the claims in the manuscript are too strong and do not accurately represent the results. In particular (1) many claims are specific to the thresholds that are used, with BLAZE using a more conservative threshold than Sockeye. The authors show that the conservative threshold appears to be more useful, but this does not indicate that the method in BLAZE is more accurate. (2) Evaluation of barcode detection methods should be primarily based on the ability to detect barcodes, not on the quality of the cells associated with the barcodes at a specific threshold, which some users might prefer to address through filtering of cells based on expression data. Specific examples and comments

* In this work it is claimed "BLAZE outperforms existing tools". The majority of the results compare BLAZE and Sockeye at one set of parameters. At these parameters BLAZE is shown to be more conservative which leads to clearer outcomes in the clustering. However this does not prove BLAZE is outperforming Sockeye on the task of barcode detection, as Sockeye may be able to achieve these same results with more stringent thresholds. When the thresholds are compared in Fig.6 a minor increase in the AUC is seen for BLAZE. It is possible that BLAZE only outperforms Sockeye for thresholds that are lower than BLAZE's default parameters. Therefore, there are no results to show that in practice BLAZE outperforms Sockeye when comparable thresholds are used.

* To address this, the authors should update Figure 6 to highlight the point on each line that corresponds to the default thresholds for BLAZE and Sockeye. Furthermore Figure 6 should be shown as the full range of recall, with the zoomed in version as an addition. The current visualisation is helpful, but overstates the difference between methods.

* Depending on these results, the authors should adjust their claims.

* Figure 2/Table 2 could be reproduced using more conservative and comparable parameters for Sockeye. This indicates that a conservative threshold is useful, which is valid for both Sockeye and BLAZE

We thank the reviewer for these useful suggestions. As outlined in our next response below we have adjusted our claims to ensure accuracy. Secondly as outlined above to reviewer 2, due to read mis-assignment to non-cell associated or uninformative barcodes we believe an accurate barcode whitelist is a better strategy than filtering out such barcodes based on expression.

We have taken several steps to address concerns about BLAZE - Sockeye comparisons only involving one set of parameters and feel these changes have improved the robustness of the evaluation of BLAZE compared to Sockeye.

As reviewer 3 suggested we have updated figure 6 (now figure 7 in the revised version) to highlight the default threshold(s) utilised by each method and added S8A to include the full range of recall. These show BLAZE has higher precision over a wide range of recall values.

The thresholding in both tools is automatically generated and we believe that most users would run these tools with their default thresholding strategies. Therefore, we believe it is fair to primarily assess BLAZE and Sockeye using default settings. While we agree testing more conservative thresholds for Sockeye would be useful, this is not a user definable parameter for the Sockeye pipeline and there is no documentation for how to alter it. In contrast, thresholds are adjustable parameters in BLAZE. To further enable users to use different thresholds we have now created a High Sensitivity (HS) mode for BLAZE, which also allows us to test its performance using this different threshold. These results are included in Table 2 and Figures 6 & S7 and confirm BLAZE generally outperforms Sockeye. We also have incorporated simulation data to test the full range of precision and recall with a known ground truth dataset which further verifies our claims.

* The authors claim that "[BLAZE] provides a more accurate representation of the cells present in LR scRNA-seq than using matched short-reads". There are no results that evaluate the performance of BLAZE against Cell Ranger. The cells/barcodes that are detected by Cell Ranger may represent true cells (albeit low quality ones) and therefore the comparison of BLAZE swith cell ranger does not indicate higher accuracy for the task of identifying barcodes, but that BLAZE has a more stringent criteria for barcode filtering which removes barcodes that represent cells of low quality. Either additional results need to be provided that compare Blaze against matched short-reads that show it is indeed more accurate or this claim needs to be adjusted.

We understand the reviewers concerns and have adjusted the language and claims made throughout the manuscript, including the sentence quoted above. We hope it is now clearer we don’t claim anything is wrong with Cell Ranger per-se, but that barcodes from a short-read dataset will not be an optimal match for a long-read dataset. In this context we do perform a number of comparisons between the barcodes identified by Cell Ranger and BLAZE and analyses such as Empty Droplets and clustering of the scmixology2 5 cell lines clearly show some barcodes from Cell Ranger do not denote true cells in the long-read data. We now generally now refer to these as “uninformative cells” or “non-cell associated barcodes” as a large proportion of these contain an ambient RNA profile and/or have low support in the LR data.

2) Usability of BLAZE

The usability of BLAZE is an important factor. Currently the authors represent the runtimes using 32 cores. Could the authors provide more information on running BLAZE with less cores and the memory requirements. A quantified comparison of the memory usage and run time to do the full workflow with Cell Ranger and Sockeye at the same specifications is also important to support BLAZE despite it not being a fair comparison due to the additional tasks in Sockeye. The authors could compare the performance of BLAZE to using this python script if they are concerned about an unfair comparison (<https://github.com/epi2me-labs/wf-single-cell/blob/master/subworkflows/process_bams.nf>)

We thank the reviewer for this comment and agree that runtimes with 32 cores may not be applicable to users with limited processing power. We have addressed the concerns of the reviewer by testing BLAZE again on all data sets using 6 CPUs and have added runtime information to **Additional file 5**. In addition, we have updated the software and now the memory requirements for BLAZE are significantly reduced and it automatically performs multiprocessing even with a single fastq file, which makes it much faster and more efficient.

We note that Sockeye identifies cell barcodes from a mapped file while BLAZE does not and therefore a fair runtime comparison between the two methods is not really possible. The python script above will only generate a whitelist assuming reads have already been mapped.

Minor Comments:

* Sockeye is deprecated and replaced: [**https://github.com/epi2me-labs/wf-single-cell**](https://github.com/epi2me-labs/wf-single-cell)

As reviewer 3 has noted, Sockeye has been depreciated. As outlined by the Sockeye lead programmer John Beaulaurier, this occurred as a direct consequence of BLAZE being made publicly available on bioRxiv in August 2022. Oxford Nanopore technologies have since begun developing wf-single-cell (a pipeline which includes an updated version of Sockeye) and have incorporated BLAZE’s qscore filtering step and quantile-based approach into the workflow (including our thresholds). As such the ONT wf-single-cell workflow is now largely based on BLAZE for the barcode identification and whitelist generation step of the workflow.

We have noted this in the discussion:

“As a consequence of the BLAZE software and pre-print becoming publicly available prior to publication, Sockeye has been depreciated and BLAZE’s qscore filtering step and quantile-based approach incorporated into ONTs replacement software pipeline wf-single-cell.”

* BLAZE is linked to FLAMES. Would it be an option to create an output that other tools such as StringTie2 (used in Sockeye) could use (eg. de-multiplexed fastq files). That could increase adaptation of BLAZE since most downstream tools for long read single cell methods are not yet established.

We agree a more cross compatible output file would increase the usability of BLAZE and other single cell tools. Currently BLAZE outputs a barcode whitelist in the same csv file format as Cell Ranger, which is compatible with all independent software for 10x ONT LR scRNA-seq we are aware of. It would also be simple for any newly developed downstream tools to take as input.

Regarding tools such as StringTie2 or FLAIR, these require a mapping file as input and also don’t perform other steps required for single cell analysis (i.e.: UMI calling and clustering). Enabling direct BLAZE compatibility with these tools would require BLAZE to perform a number of additional functions, which are beyond the scope of our current work and would duplicate steps performed by FLAMES. If users wish to use these other existing tools it is possible to generate a barcode whitelist with BLAZE and perform mapping with other tools. The subsequent BAM file could be input into StringTie2 or FLAIR.

* In the evaluation the authors use data from Tian et al (2021) where barcodes were assigned to specific cell lines. It would be beneficial to readers for the authors to briefly summarise how barcodes were assigned to each cell line, as the method could have intersected with one of the applied tools creating a bias (it doesn't, but unless a reader looks up another paper the uncertainty is there).

We have added this additional information to the methods section of the updated manuscript to better explain how barcodes were assigned to cell lines by Tian et al:

“To determine the cell line for each barcode Tian *et al.* (2019) used Demuxlet55 [42], which uses the genetic variation between cell lines to identify the most probable identity of each barcode [43].

* "These results suggested that Cell Ranger and Sockeye may be consistently identifying false positive long-read barcodes" - As Cell Ranger does not detect false positive long-read barcodes, this is incorrect to say.

We thank reviewer 3 for noticing this error. We have removed this sentence from the updated manuscript and replaced it with:

“The larger number of cell barcodes identified by Sockeye compared to the number of cells sequenced further suggested it is identifying non-cell associated barcodes.”

- There are several observations made when comparing BLAZE with Cell Ranger and Sockeye and it would be good if the authors could speculate as to their origin in either the results of discussion:

* The authors suggest the higher numbers of white-listed barcodes in Cell Ranger may be because these cells exist in SR data but are poorly represented amongst long-reads. Can the authors speculate why this would happen? If the reason is due to sequencing depth, why would there not be a change in the number of accepted barcodes between the PromethION and GridION runs? This could be partially explained with Barcode rank plots (knee plots) of the GrioION and Q20 runs.

* BLAZE and Sockeye find 5 barcodes that are not in the short read data. Could the authors speculate on how this could occur?

As BLAZE performs quartile-based thresholding it is robust to changes in read depth and therefore we don’t believe that the number of reads should significantly impact the barcode whitelist (See Table 2, and section “Barcode detection with BLAZE is robust to changes in read depth or read accuracy”).

We have added some of our ideas regarding the additional barcodes that are detected by Cell Ranger to the discussion:

“Conversely, >20% of barcodes identified by Cell Ranger were not found by BLAZE default mode. These barcodes were supported by few long-reads, expressed comparatively few genes and isoforms and often had an ambient RNA profile. We hypothesise that despite sequencing matched samples, some cells (and hence barcodes) found in SR data are poorly represented amongst the long-reads. We theorise that the separate PCR reactions in the long- and short-read library preps, either by chance and/or bias, led to some cells dropping out of the libraries. Supporting this, the Cell Ranger knee plot showed that barcodes not found by BLAZE had low UMI counts in the SR data. Such barcodes are the most likely not to be found in matched LR sequencing due to chance, biases or differences in read depths. Consequently, the use of long-read only barcode identification methods should produce whitelists that more faithfully represent cells profiled with long-read sequencing.”

The 5 barcodes that are found by BLAZE and Sockeye but not by cell Ranger are well supported in both the short and long read data sets. We cannot confidently say why they have not been included by Cell Ranger considering the short read support and their position on the barcode rank plot (**Figure 1D**), which appears to be above the Cell Ranger threshold. We speculate Cell Ranger has called these cells as empty droplets based on their expression profile and excluded them from the barcode whitelist. Of these 5 barcodes 2 are classed as empty droplets in our analysis providing some support for this speculation.

**Second round of review**

**Reviewer 1:** The authors have satisfied my reservations and it now reads really well and the authors should be congratulated on the changes they made. I have only one minor comment and that relates to the github code not being documented for the updated high sensitivity mode. Before publication appropriate documentation and release of the code should be performed

**Reviewer 2:** The authors do a great job addressing my comments and concerns. I specifically would highlight them:

-Testing BLAZE on a small read coverage per cell dataset

-Testing BLAZE on a simulation data with known ground truth

-Expanding their analysis of the barcodes detected by Sockeye and/or Cell Ranger but not BLAZE using Empty Droplet analysis and attempting a differential gene expression analysis

-Improving BLAZE's software for better multithreading and significantly reduced memory usage. I re-tested BLAZE myself on an inhouse dataset and it performed similarly to what the authors state in terms of memory and time.

**Reviewer 3**

In my reviewer's comments, I outlayed a few concerns on the data comparing BLAZE to Sockeye and CellRanger.

To address the concern in the BLAZE-Sockeye comparison, where both tools were producing results at different sensitivity and precision thresholds and therefore not comparable, the authors added a high sensitivity mode to Sockeye. With the high sensitivity mode, BLAZE was able to achieve a comparable or greater sensitivity to Sockeye without sacrificing precision on both their data and newly produced simulated data. Furthermore, using this mode they showed they could capture the same empty-droplet filled clusters as the other tools that were previously discarded by BLAZE, if this is something desired by the user.

In the original manuscript, the authors claimed that BLAZE provides an accurate representation of the cells present in the data, when instead the presented data suggests that they provide a representation of high quality barcodes. In the revised manuscript, the authors have changed their claim accordingly to better reflect the data and results (in many places, but not the abstract, where "barcodes" might still be more appropriate than "cells"). Furthermore, the authors provide evidence that barcodes filtered out by BLAZE either belong to uninformative cells or are not associated with a cell.

All minor comments were addressed sufficiently. Together, with the new additions to the paper, it now robustly supports the author's claims that BLAZE accurately and efficiently identifies high-quality barcodes better than its contemporary tools.
